# The relationship between patient empowerment and related constructs, affective symptoms and quality of life in patients with type 2 diabetes: a systematic review and meta-analysis

**DOI:** 10.3389/fpubh.2023.1118324

**Published:** 2023-04-17

**Authors:** Andrea Duarte-Díaz, Lilisbeth Perestelo-Pérez, Amado Rivero-Santana, Wenceslao Peñate, Yolanda Álvarez-Pérez, Vanesa Ramos-García, Himar González-Pacheco, Libertad Goya-Arteaga, Miriam de Bonis-Braun, Silvia González-Martín, Yolanda Ramallo-Fariña, Carme Carrion, Pedro Serrano-Aguilar

**Affiliations:** ^1^Canary Islands Health Research Institute Foundation (FIISC), Tenerife, Spain; ^2^Department of Clinical Psychology, Psychobiology and Methodology, Universidad de La Laguna, (ULL), Tenerife, Spain; ^3^Network for Research on Chronicity, Primary Care, and Health Promotion (RICAPPS), Madrid, Spain; ^4^The Spanish Network of Agencies for Health Technology Assessment and Services of the National Health System (RedETS), Madrid, Spain; ^5^Evaluation Unit (SESCS), Canary Islands Health Service (SCS), Tenerife, Spain; ^6^Multiprofessional Unit of Family and Community Care of La Laguna-Norte, Tenerife, Spain; ^7^eHealth Lab Research Group, School of Health Sciences, Universitat Oberta de Catalunya (UOC), Barcelona, Spain

**Keywords:** empowerment, diabetes, affective outcomes, quality of life, systematic review and meta-analysis, self-efficacy

## Abstract

**Introduction:**

The aim of this systematic review is to assess the relationship between patient empowerment and other empowerment-related constructs, and affective symptoms and quality of life in patients with type 2 diabetes.

**Methods:**

A systematic review of the literature was conducted, according to the PRISMA guidelines. Studies addressing adult patients with type 2 diabetes and reporting the association between empowerment-related constructs and subjective measures of anxiety, depression and distress, as well as self-reported quality of life were included. The following electronic databases were consulted from inception to July 2022: Medline, Embase, PsycINFO, and Cochrane Library. The methodological quality of the included studies was analyzed using validated tools adapted to each study design. Meta-analyses of correlations were performed using an inverse variance restricted maximum likelihood random-effects.

**Results:**

The initial search yielded 2463 references and seventy-one studies were finally included. We found a weak-to-moderate inverse association between patient empowerment-related constructs and both anxiety (*r* = −0.22) and depression (*r* = −0.29). Moreover, empowerment-related constructs were moderately negatively correlated with distress (*r* = −0.31) and moderately positively correlated with general quality of life (*r* = 0.32). Small associations between empowerment-related constructs and both mental (*r* = 0.23) and physical quality of life (*r* = 0.13) were also reported.

**Discussion:**

This evidence is mostly from cross-sectional studies. High-quality prospective studies are needed not only to better understand the role of patient empowerment but to assess causal associations. The results of the study highlight the importance of patient empowerment and other empowerment-related constructs such as self-efficacy or perceived control in diabetes care. Thus, they should be considered in the design, development and implementation of effective interventions and policies aimed at improving psychosocial outcomes in patients with type 2 diabetes.

**Systematic review registration:**

https://www.crd.york.ac.uk/prospero/display_record.php?ID=CRD42020192429, identifier CRD42020192429.

## 1. Introduction

Diabetes Mellitus (DM) is a major public health problem with a high and increasing frequency (1). According to the IDF Diabetes Atlas for 2021, an estimated 536.6 million individuals between the ages of 20 and 79 were diagnosed with diabetes, and this number is projected to rise to 783.2 million by 2045 (2). Type 2 Diabetes Mellitus (T2DM), accounts for 90–95% of all diagnosed cases of DM (3, 4). T2DM imposes a considerable burden on patients' health and health-related quality of life (HRQOL), as well as on socioeconomic issues (5, 6).

Although there is no cure for T2DM, it is possible to improve disease control to delay clinical complications and mortality by means of personalized and complex therapeutic strategies including lifestyle modification (7). Adherence to self-care behaviors is not easy but it is needed to achieve sustained long-term control and improve health outcomes. Both psychological and psychosocial problems have been identified as common barriers to self-care in patients with T2DM (8).

Research studies have reported a higher incidence of T2DM in individuals with major depression compared to the general population (9). Nearly one in four patients with T2DM suffer from comorbid depression (10). Besides, compared to the general population, a higher incidence and prevalence of anxiety disorders have also been reported among people with T2DM (11, 12). According to a recent meta-analysis on the prevalence of T2DM in mental disorders, 14% of individuals with anxiety disorder and 9% of individuals with depression have been diagnosed with T2DM (13). It is reported that patients with T2DM and comorbid mental health problems are more likely to have diabetes complications (14) and are less likely to meet the guidelines for a healthy lifestyle and self-care recommendations (15–17), with greater difficulties in achieving and maintaining diabetes control. It has recently been pointed out that T2DM patients with comorbid anxiety or depressive disorders have a higher likelihood of visiting the emergency room when compared to diabetic patients without mental health problems (18). Furthermore, the risk of 4-year-all-cause mortality is 14% higher in those T2DM patients with comorbid depression (18). Depression and chronic psychological stress can trigger the activation of the hypothalamic-pituitary-adrenal axis, which in turn stimulates the sympathetic nervous system, increases platelet aggregation response, and inflammation while reducing insulin sensitivity (19, 20). These physiological changes may contribute to poor glycemic control and an increased risk of diabetes-related complications such as retinopathy, enteropathy, dermatopathy, diabetic foot, and neuropathy (21).

Understanding mental health issues in patients with T2DM has become a major concern. The American Diabetes Association highlight the need to integrate psychosocial care with person-centered medical care for people with diabetes to optimize health outcomes and promote HRQOL (22). Person-centered care (PCC) has a holistic view of patient care, focusing on the need of seeing people beyond the illness, valuing their needs and respecting their rights and dignity. One of the core elements of PCC is patient empowerment, defined as a process through which people can gain control over decisions and actions affecting their health (23). From this point of view, the aim of patient empowerment is to provide them with critical thinking, skills and tools to take responsibility for their health and wellbeing, develop autonomy and make informed autonomous decisions.

Despite the growing interest in PCC and the encouragement of patients to be actively involved in their care, defining and measuring empowerment is still challenging. Firstly, it has not only been conceptualized as a process but also as an outcome. It can be considered as the process through which patients gain control over their healthcare and it can be achieved through patient-centeredness. Accordingly, patient empowerment has been defined as a meta-paradigm that connects patient participation and patient-centeredness (24). From this point of view, patient participation may be considered a condition to achieve PCC, which in turn can promote patient empowerment (24). Moreover, it can also be considered as an outcome since patients are empowered when they have the necessary knowledge and skills to influence their own behavior to improve their quality of life (QoL) (25). Secondly, most definitions of patient empowerment include references to other theoretically-related constructs such as self-efficacy, patient activation or perceived control (26–28). Even when there have been attempts to clarify the boundaries between empowerment and these concepts, it may be difficult to fully differentiate one from the other (26). Bravo et al. (29) proposed a novel conceptual map of patient empowerment in 2015. According to the authors, patient empowerment can be conceived as a state ranging across a spectrum from low to high levels and depends on patient, provider and healthcare system factors. In addition, they suggested that this level of patient empowerment can be potentially measurable using a set of related constructs as indicators, including those referring to patients' capacities (e.g., self-efficacy, perceived control) and behaviors (e.g., patient activation) (29). Empowerment is an umbrella term, but the core of this concept is the idea of supporting patients to become more responsible for their own health (30). Patient empowerment is thus a more wide-ranging and multidimensional concept (31), but there's no universally accepted instrument that can be used to measure it (32).

Promoting the participation of people in their own healthcare is considered an ethical imperative included in the Declaration of Salzburg (33). Previous studies have shown that patients with Type 2 Diabetes Mellitus (T2DM) who report higher levels of patient empowerment tend to experience fewer affective symptoms (34, 35). Furthermore, in a secondary analysis of a randomized controlled trial it was found that an increase in patient empowerment is associated with improvements in both anxiety and depressive symptoms (36). This finding is particularly significant considering that affective and emotional disorders in T2DM have been associated with non-adherence to diet, physical activity and medication (16, 37, 38). Existing evidence suggests that empowering patients may not only improve affective symptoms but also enhance QoL (39, 40). Two systematic reviews (SR) have shown that empowerment-based strategies may improve clinical, behavioral and psychological outcomes in patients with T2DM (41, 42). Baldoni et al. (41) reported that programs based on collective empowerment strategies lead to an increase in confidence and DM knowledge, better attitudes toward the disease and more healthy eating patterns. Likewise, Aquino et al. (42) concluded that individual empowerment-based strategies have several psychosocial benefits such as more self-care behaviors, increased motivation, self-efficacy as well as DM knowledge and better QoL.

Even though previous SR have highlighted the effectiveness of empowerment-based interventions, to the best of our knowledge no previous reviews have synthesized data on the association between the level of patient empowerment or empowerment-related constructs and affective outcomes and QoL in patients with T2DM. This SR and meta-analysis (MA) address this gap and provide additional and relevant information on the relationship between patient empowerment itself and affective symptoms and QoL while also highlighting the importance of other empowerment-related constructs.

## 2. Methods

### 2.1. The protocol and registration

The results of this SR and MA were reported based on the Preferred Reporting Items for Systematic reviews and Meta-Analysis (PRISMA) 2020 statement (43) (Supplementary material 1). This SR was registered in the International Prospective Register of Systematic Reviews (PROSPERO) with the registration number CRD42020192429.

### 2.2. Eligibility criteria

#### 2.2.1. Participants

Studies addressing patients with T2DM, aged 18 years or older were included. Studies involving children or adolescents, patients with type 1 DM, gestational DM or participants with pre-diabetes were excluded.

#### 2.2.2. Outcomes

**Figure 1:**
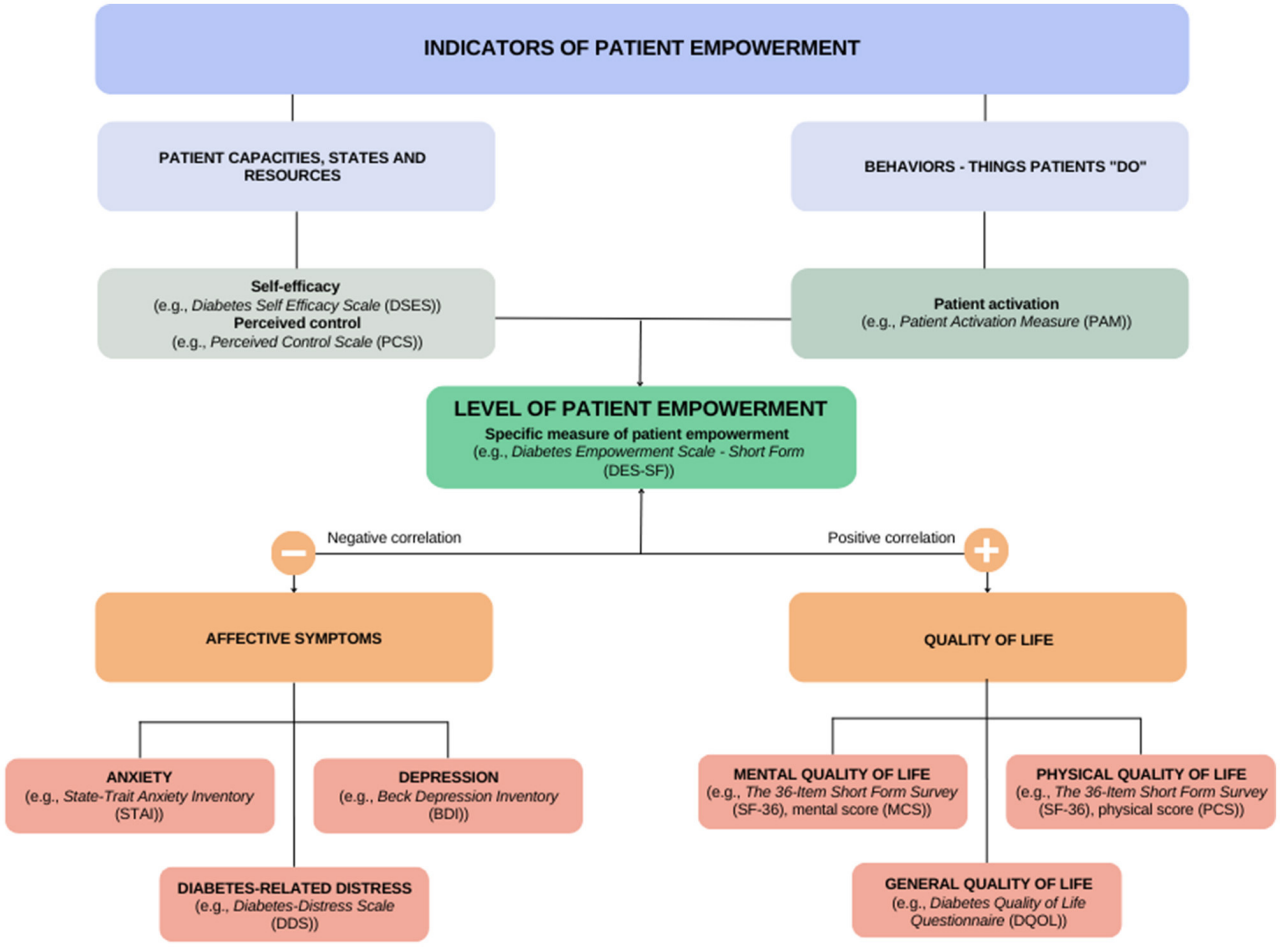
Diagram of patient empowerment and related-constructs in relation to affective symptoms and quality of life. Adapted with permission from Bravo et al. (29), licensed under CC BY 4.0.

Studies analyzing the association between patient empowerment or empowerment-related constructs and both affective symptoms (i.e., anxiety, depression and distress) and QoL were included. Given the lack of consensus on the definition of patient empowerment, and in order to be exhaustive, this review included not only studies assessing patient empowerment itself, but also other empowerment-related constructs that might be potential indicators, such as self-efficacy, patient activation and perceived control. Outcomes related to self- or clinician-rated symptoms of anxiety, depression and distress, as well as self-reported QoL, using validated instruments, were included. Figure 1 shows the anticipated theoretical relationships among the variables evaluated in the present review.

#### 2.2.3. Study design

Since terms of associations can be reported in various study designs, we included all study designs in the eligibility criteria. Clinical trials, pre-post intervention studies or observational studies addressing the association of patient empowerment or empowerment-related constructs on affective outcomes (i.e., anxiety, depression or distress) and QoL were subsequently included. Clinical trials not including a specific measure of patient empowerment, self-efficacy, patient activation or perceived control were excluded, even if the intervention was based on PCC principles. In anticipation of not finding much longitudinal evidence, cross-sectional studies were also considered to test the hypothesis of no association between the study variables. Conference abstracts, letters, commentaries, essays, book chapters, qualitative studies, protocols and reviews were also excluded.

### 2.3. Study selection and data extraction

The following electronic databases were consulted from inception to July 2022: Medline, Embase, PsycINFO and Cochrane Library. To ensure comprehensiveness, the search strategy included keywords related to patient empowerment as well as other related constructs that could serve as potential indicators of the level of patient empowerment. The following terms were used individually and combined according to the Medical Subject Heading (MeSH) terms: “diabetes,” “anxiety,” “depression,” “quality of life,” “empowerment,” “self-efficacy” and “patient activation” (Supplementary material 2). No language or publication year restrictions were applied to limit the search. Monthly Medline searches were conducted until the study submission. Additionally, the list of references of all eligible articles were screened and manual searches in Google Scholar were undertaken.

### 2.4. Study selection and data extraction

All citations extracted from the different electronic databases were imported into a standardized Microsoft Excel data sheet and duplicates were removed. Firstly, two members of the research team independently reviewed all titles and abstracts in order to pre-select those meeting the inclusion criteria. Secondly, the full-text of the potentially relevant studies was screened for eligibility by two reviewers. Any disagreement was solved by discussion and consensus and a third reviewer was consulted if needed. From each included study, two reviewers independently extracted data on the following variables according to a standardized data extraction form in Microsoft Excel: first author, year of publication, country, number of participants, mean age, study design, study population, outcomes, effect estimates for the main outcomes and main results.

### 2.5. Risk of bias assessment

The Joanna Briggs Institute critical appraisal checklist was used (112) was used to evaluate the quality of cross-sectional studies. Version 2 of the Cochrane risk-of-bias tool for randomized trials (113) was used to assess the risk of bias in randomized trials. The methodological quality of cohort and pre-post studies was assessed using the National Institute of Health quality assessment tools (114). Quality assessment was undertaken by two independent reviewers and disagreements were solved by discussion and consensus or after consulting a third reviewer.

### 2.6. Statistical analyses

Meta-analyses were performed using the transformation of r values into Fisher's z scores and then reconverting them to r values. The associations were classified as weak (*r* = 0.10 to 0.29), moderate (r = 0.30 to 0.49) or strong (≥50) (115). When effect sizes different to Pearson's correlation were reported, each was converted using the following formulas:

(1) Spearman's correlation to Pearson's correlation (116):

$$\begin{array}{l} r=2*\sin\left(\frac{\;\rho^{*}\pi }{6}\right)\; \end{array}$$

(2) Regression coefficient from multiple linear regression to Pearson's correlation (117):

$$\begin{array}{l} r=\;\beta +0,05\lambda \; \end{array}$$

(3) χ2 to Pearson's correlation (118):

$$\begin{array}{l} r=\sqrt{\;\frac{\chi^{2}}{n}}\; \end{array}$$

Where n denotes sample size, λ = 1 if β is positive and λ = 0 if β is negative.

When multiple correlation coefficients were reported in a particular study (i.e., the association between different empowerment subscales and general QoL), an average effect size was calculated in order to preserve statistical independence between samples. Heterogeneity was calculated by means of the Cochran's Q test and quantified by Higgins I^2^ statistics (119). Correlation coefficients were pooled using an inverse variance restricted maximum likelihood random-effects MA (120), with 95% as confidence interval (95%CI) and visually displayed through forest-plots. The authors conducted subgroup analyses for categorical variables and bivariate meta-regression for continuous variables in addition to using random-effects models when significant heterogeneity was present. Additionally, a 'leave-one-out' sensitivity analysis was performed to evaluate the influence of potential outliers on the pooled effect estimate and to explore other sources of heterogeneity. The available data allowed the analysis of the influence of three moderating variables pre-specified in the study protocol: type of empowerment-related construct, mean age and Hba1c. Other subgroups and potential moderators were subsequently defined p*ost-hoc*: type of study (cross-sectional vs longitudinal), type of distress measure (diabetes-related distress vs. general distress), gender (defined as % of females) and years since diagnosis. Subgroup analyses were performed if at least two studies for each subgroup of interest were available. When at least ten correlation coefficients were included, the publication bias was evaluated using the Egger test (121) and the trim-and-fill method (122) was used to correct for possible funnel plot asymmetry. All the analyses were performed in Stata v17 (123) using the *meta* package (124).

## 3. Results

**Figure 2:**
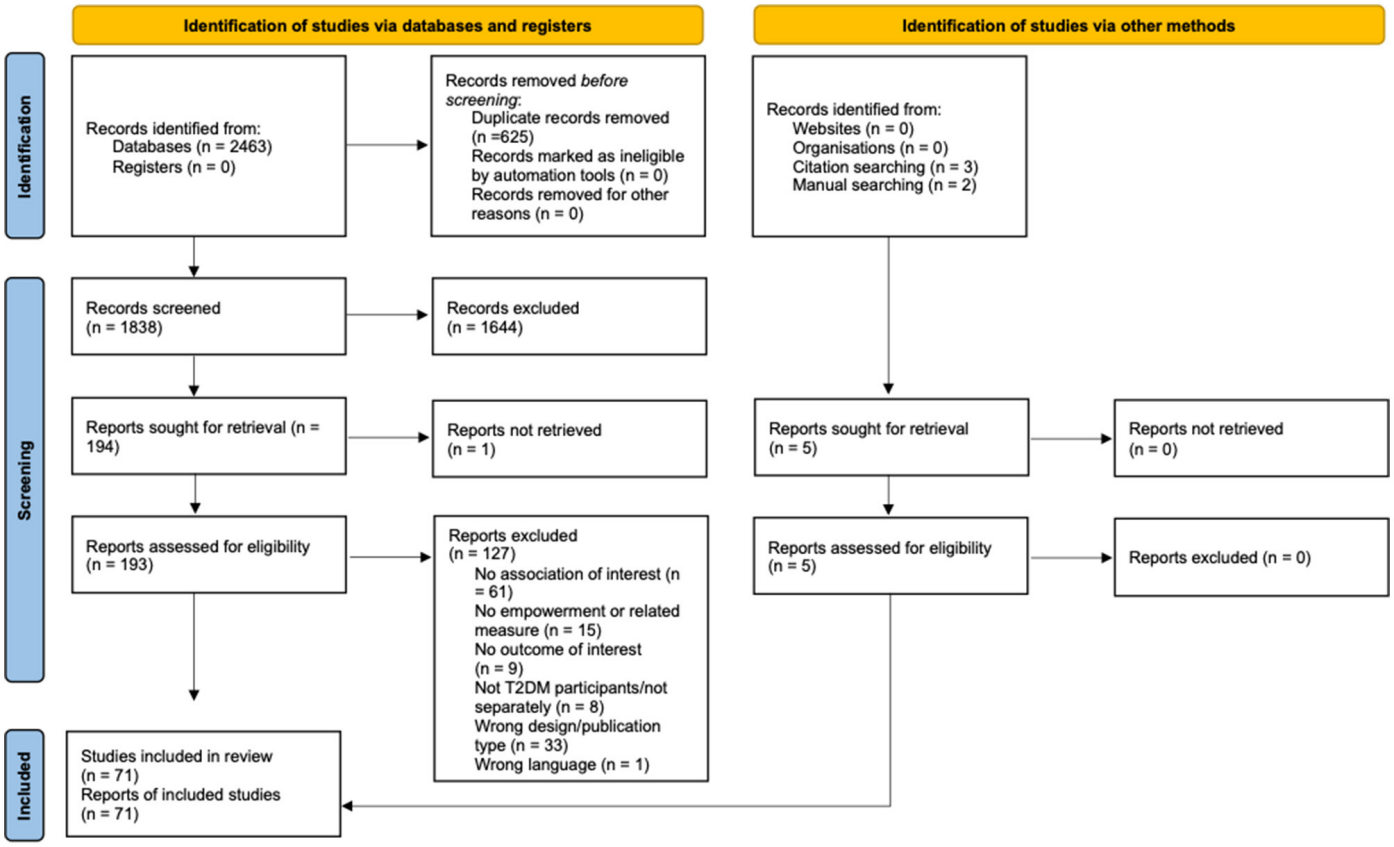
PRISMA flow-chart of the study selection process.

The initial search in the electronic databases yielded 2,463 references. After removing duplicates, 1557 records were screened by title and abstract and 191 full-text articles were assessed for eligibility. Five additional records were identified through manual searches (50, 80) and citation list (62, 104, 110). Seventy-one studies were finally included: sixty-one cross-sectional studies (34, 35, 44, 45, 47–69, 71–78, 80, 83–88, 91–97, 99–102, 104–111), five observational prospective studies (36, 70, 81, 82, 89), four randomized controlled trials (RCTs) (79, 90, 98, 103) and one pre-post intervention study (46). Figure 2 shows the flowchart of the selection process of the studies'.

### 3.1. Characteristics of the included studies

The total sample was made up of 20,825 participants, mean age ranging from 46 to nearly 74 years of age and 57% were females. Thirty-two studies were carried out in Asia, twenty-three in North America, thirteen in Europe, two in Africa and the remaining two in South-America. Table 1 shows the studies' characteristics.

**Table 1:** Main characteristics of the included studies.

**Author, year**	**Country**	**Design**	**Sample size**	**Mean age**	**% of females**	**Empowerment/ indicator scale**	**Outcomes**			
							**Anxiety**	**Depression**	**Distress**	**QoL**
**Patient empowerment**										
Ababio et al. (44)	Ghana and Nigeria	Cross-sectional	396	NR	71.75	DES-SF	-	-	-	WHOQOL-BREF
Ching et al. (45)	Malaysia	Cross-sectional	151	55 ± 13	33.8	DES-SF	-	-	-	DQOL
Clarke et al. (46)	Ireland	Pre-post^a^	392	64 ± 10.2	41	DES-SF	-	-	-	WHO-5
Duarte-Díaz et al. (36)	Spain	Prospective^b^	2334	55.70 ± 7.1	51.9	DES-SF	STAI-S	BDI-II	-	-
Hernández et al. (latin) (47)	USA	Cross-sectional	116	54.7	71.4	DES-SF	-	PHQ-9	-	-
Hernández et al. (african) (47)	USA	Cross-sectional	134	51.8	65.8	DES-SF	-	PHQ-9	-	-
Lin et al. (48)	China	Cross-sectional	254	55.26	NR	DES	-	PHQ-9	DDS-17	-
Oliveira et al. (49)	Portugal	Cross-sectional	137	73.9 ± 6.7	46.7	DES-SF	-	-	-	EQ5D
Simonsen et al. (50)	Finland	Cross-sectional	2630	63 ± 8	44	DES-SF	-	-	Self-developed	-
Sympa et al. (34)	Greece	Cross-sectional	170	66.71 ± 11.23	52.35	DES-SF	-	GHQ-28	-	-
Wang et al. (51)	Taiwan	Cross-sectional	428	58.31 ± 10.55	50.7	DES-SF	-	-	DDS-17	DQOL
Zhu et al. (52)	China	Cross-sectional	397	56.33 ± 24.89	41.1	DES-SF	-	-	-	WHOQOL-BREF
**Self-efficacy**										
Abdelgaffar et al. (53)	Tunisia	Cross-sectional	100	58.1 ± 8.5	33	DMES	-	-	PAID-5	-
Al Amer et al. (54)	Jordan	Cross-sectional	220	58.2 ± 10.8	52.3	DMSES	-	PHQ-9	-	-
Al Dwaikat0 et al. (55)	Jordan	Cross-sectional	339	59.6 ± 11.1	56.6	DMSES	DASS-21	DASS-21	DASS-21	-
Alipour et al. (56)	Iran	Cross-sectional	80	46	100	SGSES	-	-	DASS-21	ADDQoL
Alzubaidi et al. (57)	Dubai	Cross-sectional	696	59.1 ± 8.9	48.6	Two items	-	-	DDS-17	-
Anderson et al. (58)	USA	Cross-sectional	117	57.44 ± 9.83	42.7	MDQ	-	PHQ-9	PAID-5	-
Aoto et al. (59)	Philippines	Cross-sectional	117	64.48	76.9	DSES	-	-	-	SF-8
Azadbakht et al. (60)	Iran	Cross-sectional	519	69.39 ± 6.78	53.2	DSES	-	-	DDS-17	-
Azami et al. (61)	Malaysia	Cross-sectional	142	56 ± 11.1	65.5	DMSES	-	CES-D	-	-
Chao et al. (62)	USA	Cross-sectional	445	56.3 ± 11.4	50.1	Single item	-	PHQ-8	-	-
Cherrington et al. (63)	USA	Cross-sectional	162	56 ± 11.48	60.5	PDSMS	-	CES-D	-	-
Chew et al. (64)	Malaysia	Cross-sectional	338	60.6 ± 10.1	55.7	DMSES	-	-	DDS-17	-
Coffman et al. (65)	USA	Cross-sectional	115	69.3 ± 7.85	62.6	DMSES	-	CES-D	-	-
Devarajooh et al. (66)	Malaysia	Cross-sectional	371	54.7	62	DMSES	-	PHQ-9	DDS-17	-
Emery et al. (67)	USA	Cross-sectional	78	63.10 ± 9.53	56	DSES	-	-	PAID-5	SF-12
Fereydouni et al. (68)	Iran	Cross-sectional	496	55.9 ± 9.62	75.8	Van der Bijl's scale	-	-	-	Thomas's QoL questionnaire
González et al. (69)	USA	Cross-sectional	142	55.95 ± 9.24	44.4	SSES	-	MADRS	DDS-17	-
Hsu et al. (70)	Taiwan	Prospective^c^	185	55.57 ± 11.06	38,4	SEIS	-	-	-	DSQOLS
Huang et al. (71)	USA	Cross-sectional	155	69.07 ± 10.75	52.9	DMSES	-	CES-D	DDS-17	-
Huayanay-Espinoza1 et al. (72)	Peru	Cross-sectional	123	61.8 ± 11.1	65.9	GSES	-	-	-	D39
Indelicato et al. (73)	Italy	Cross-sectional	172	64 [58-69]	39,5	MDQ	BAI	BDI-II	-	-
Jahanlou et al. (74)	Iran	Cross-sectional	256	49.16 ± 9.5	67.5	SEQ	-	-	-	WHOQOL-BREF
Ji et al. (75)	China	Cross-sectional	207	56.1 ± 11.4	50.2	SE-T2DM	-	CES-D	-	-
Ji et al. (76)	China	Cross-sectional	304	64.1 ± 10.3	57.2	SE-T2DM	-	-	SDS	-
Kav et al. (77)	Turkey	Cross-sectional	200	60.5 ± 9.7	61	SES	-	BDI	-	
Kim et al. (78)	Korea	Cross-sectional	198	NR	40.4	IMDSES	-	GDSSF-K	DDS-17	-
Kim (79)	USA	RCT^d^	209	58.7 ± 8.4	40.9	CDSEPS	-	-	-	DQOL
Kobling et al. (80)	Hungary	Cross-sectional	250	59.2 ± 13.6	56.8	DSES	-		-	SF-36
Latham and Calvillo (81)	USA	Prospective	240	41.63 ± 11.16	NR	DSES	-	-	-	DQOL
Latham and Calvillo (82)	Mexico	Prospective	109	47.5	80	DSES	-	-	-	DQOL
Lin et al. (35)	Taiwan	Cross-sectional	198	51.2 ± 11.0	37,4	DMSES	HADS	HADS	PAID-C	
Matteucci et al. (83)	Italy	Cross-sectional	154	59 ± 8	53.2	SELF-E	STAI	HADS	-	
Messina et al. (84)	Italy	Cross-sectional	165	65.2 ± 9	33.3	DMSES	-	PHQ-9	PAID-5	WHO-5
Oviedo-Gómez et al. (85)	Mexico	Cross-sectional	256	58.28 ± 10.85	75.7	MDQ	-	-	-	DQOL
Padget et al. (86)	Croatia	Cross-sectional	147	59.0	51	DSES	-	ZSDS	-	-
Park et al. (87)	Korea	Cross-sectional	150	54.1	100	SEMD	-	CES-D	-	-
Pisanti et al. (88)	Italy	Cross-sectional	184	61.3 ± 9.2	53.8	MDQ	SCL-90	SCL-90-R	-	-
Rao et al. (89)	USA	Prospective	71	52.6 ± 12.03	43.7	SEAMS	-	PHQ-9	-	-
Robertson et al. (90)	USA	RCT^e^	85	63.81 ± 7.80	2.35	DSES	DASS-21	DASS-21	DASS-21	-
Rusni et al. (91)	Indonesia	Cross-sectional	54	NR	66.7	SES	-	-	-	NI
Sacco et al. (92)	USA	Cross-sectional	56	54	55	MDQ	-	PHQ-9	-	-
Sacco et al. (93)	USA	Cross-sectional	99	53	54	MDQ	-	PHQ-9	-	-
Samuel-Hodge et al. (94)	USA	Cross-sectional	185	58.9 ± 12.2	64.9	PDDC	-	-	-	SF-36
Sari et al. (older) (95)	Indonesia	Cross-sectional	206	70 ± 4.60	70.87	SES	-	-	-	SF-36
Sari et al. (younger) (95)	Indonesia	Cross-sectional	435	55.32 ± 6.80	80.23	SES	-	-	-	SF-36
Sit et al. (96)	Hong Kong	Cross-sectional	329	65.0	48.3	DMSES	GAD-7	PHQ-9	-	-
Song et al. (97)	Korea	Cross-sectional	132	63.18 ± 8.7	69.7	DSES	-	-	-	SF-36
Steed et al. (98)	UK	RCT^d^	124	NR	NR	MDQ	-	-	-	ADDQoL
Suhaimi et al. (99)	Malaysia	Cross-sectional	127	47.1 ± 8.62	49.6	DMSES	-	-	-	WHO-5
Tol et al. (100)	Iran	Cross-sectional	140	53 ± 7.82	54,3	SED	-	-	-	ADDQoL
Walker and Gebregziabher (101)	USA	Cross-sectional	615	61.3 ± 10.9	61.6	PDSMS	-	-	-	SF-12
Walker and Smalls (102)	USA	Cross-sectional	378	NR	69.1	PDSMS	-	-	-	SF-12
Wichit et al. (103)	Thailand	RCT^f^	140	58.4	72.85	DMSES	-	-	-	SF-12
Winayhu et al. (104)	Indonesia	Cross-sectional	105	NR	70.5	DMSES	-	-	-	AsianDQOL
Wu et al. (105)	Taiwan	Cross-sectional	201	60.64 [22-93]	51.7	DMSES	BAI	BDI	-	-
Yang et al. (106)	China	Cross-sectional	199	63.34 ± 8.46	51.8	MDQ	-	PHQ-9	PAID-1	-
**Activation**										
Arvanitis et al. (107)	USA	Cross-sectional	300	63.2 ± 11	56.3	IMPACT-D	PROMIS-4	PROMIS-4	-	-
Kato et al. (108)	Japan	Cross-sectional	209	60.2 ± 10.1	16.6	PAM	-	PHQ-9	-	-
**Perceived control/management**										
Hernández-Tejada et al. (109)	USA	Cross-sectional	188	NI	71	PCQ-R15	-	-	-	SF-12
Paschalides et al. (110)	UK	Cross-sectional	184	60.9 ± 12.3	56	IPQ-control	WBQ-A	WBQ-D	-	SF-36
Williams et al. (111)	USA	Cross-sectional	591	68	50.4	PCS	-	PHQ-9	-	-

ADDQoL, Audit of Diabetes Dependent Quality of Life; AsianDQOL, The Asian Diabetes Quality of Life tool; BAI, Beck Anxiety Inventory; BDI-II, Beck Depression Inventory-II; CDSEPS, Chronic Disease Self-Efficacy Program-scale; CES-D, Center for Epidemiological Studies Depression; D39, Diabetes-39 DASS-21, Depression, Anxiety, and Stress Scale; DASS-21, Depression, Anxiety, and Stress Scale; DDS-17, 17-item Diabetes Distress Scale; DES, Diabetes Empowerment Scale; DES-SF, Diabetes Empowerment Scale Short Form; DMSES, Diabetes Management Self-Efficacy Scale; DQOL, Diabetes Quality of Life Questionnaire; DSES, Diabetes Self Efficacy Scale; DSQOLS, Diabetes Specific Quality of Life Scale; EQ-5D, European Quality of Life-5 Dimensions; GAD-7, General Anxiety Disorder scale; GDSSF-K, Geriatric Depression Scale Short Form Korean Version; GHQ-28, General Health Questionnaire; GSES, General Self-Efficacy Scale; HADS, Hospital Anxiety and Depression Scale; HADS, Hospital Anxiety and Depression Scale; IMDSES, Insulin Management Diabetes Self-Efficacy Scale; IMPACT-D, Influence and Motivation for Patient ACTivation in Diabetes care; Illness Perception Questionnaire; MADRS, Montgomery Asberg Depression rating scale; MDQ, Multidimensional Diabetes Questionnaire; NR, not-reported; PAID-C, Chinese Version of the Problem Areas in Diabetes Scale; PAID, Problem Areas in Diabetes Scale; PAM, Patient Activation Measure; PCQ-R15, Perceived Control Questionnaire; PCS, Perceived Control Scale; PDDC, Perceived Diabetes and Dietary Competence; PDSMS, Perceived Diabetes Self-Management Scale; PHQ-8, Patient Health Questionnaire-8; PHQ-9, Patient Health Questionnaire-9; PROMIS-4, The Patient-Reported Outcomes Measurement Information System; SCL-90-R, Symptom Checklist-90-Revised; SDS, Symptom Distress Scale; SEAMS, Self-efficacy for Adherence to Medication Use; SED, Stanford Self-Efficacy for Diabetes; SEIS, self-efficacy of injecting insulin scale; SEMD, Self Efficacy to Manage Disease; SES, Self-Efficacy Scale; SE-TD2M, Self-Efficacy Scale for People With Type 2 Diabetes; SELF-E, Generalized Self-Efficacy scale; SEQ, Self-efficacy questionnaire; SES, The Chronic Disease Management - Self-Efficacy Scale; SF-36, 36-Item Health Survey; SF-12, 12-Item Short Form Health Survey; SGES, Sherer's General Self-Efficacy Scale; SSES, Sarkar Self-Efficacy Scale; STAI, State-Trait Anxiety Inventory; WBQ-D, WellBeing Questionnaire; Depression subscale; WHO-5, The World Health Organization-Five WellBeing Index; WHOQOL-BREF, The World Health Organization Quality of Life-BREF scale; ZSDS, Zung Self-Rating Depression Scale.

^a^Association between change in empowerment level and change in reported quality of life.

^b^Association between baseline empowerment and anxiety/depression after 12 months.

^c^Association between baseline self-efficacy of injecting insulin and quality of life after 9 months.

^d^Association between baseline data.

^e^Association between post-intervention data.

^f^Association between change over time by baseline self-efficacy.

Eleven studies (*n* = 11) (34, 36, 44–52) specifically assessed patient empowerment using both long and short forms of the Diabetes Empowerment Scale. Moreover, the majority of the studies did not specifically investigate patient empowerment, but instead examined related constructs that may serve as indicators of the level of patient: self-efficacy (*n* = 54) (35, 53–75, 77–102), patient activation (*n* = 2) (107, 108) and perceived control (*n* = 3) (109–111). Overall, the quality of the studies included in the analyses ranged from low to moderate. Most cross-sectional studies clearly defined inclusion and exclusion criteria and all described subjects in detail. The main source of bias in these studies was the unclear identification of confounding factors. In prospective studies, the research question and objectives were clearly stated. However, none of them assessed the independent variable more than once over time. The overall bias in RCTs was high in two studies (79, 98) and unclear in another two (90, 103). The only pre-post study failed to clearly report information on different items and it was thus rated as being of poor quality (46). The full quality assessment can be found in Supplementary material 3.

### 3.2. The association between empowerment-related constructs and anxiety

Eleven studies (35, 36, 55, 73, 83, 88, 90, 96, 105, 107, 110) (*n* = 4,480) assessed the relation between three empowerment-related constructs and anxious symptoms. Eight studies used self-efficacy scales (35, 55, 73, 83, 88, 90, 96, 105) and three used empowerment (36), activation (107) and perceived control (110) measures, respectively. One study, not included in the MA due to the lack of numerical data (83), reported a negative association between self-efficacy and anxiety in their subsample of patients with T2DM.

**Figure 3:**
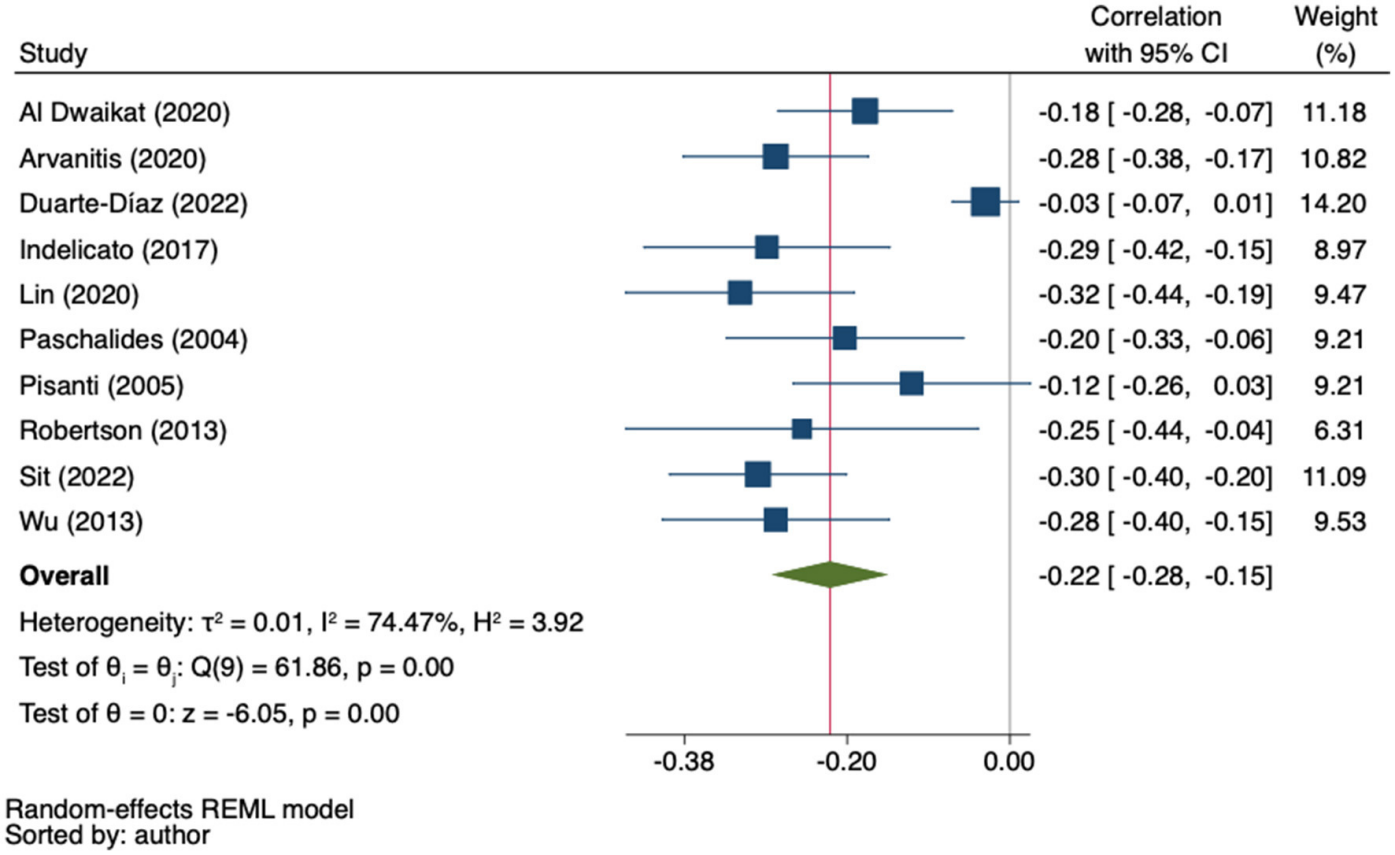
Random-effects meta-analysis on the association between empowerment and anxiety.

The MA of the remaining ten studies (*n* = 4,326) (35, 36, 55, 73, 88, 90, 96, 105, 107, 110) showed a weak inverse correlation between patient empowerment and its indicators and anxious symptomatology (*r* = −0.22; 95%CI −0.28 to −0.15; *I*^2^ = 74.47%; *k* = 10) (Figure 3).

**Figure 4:**
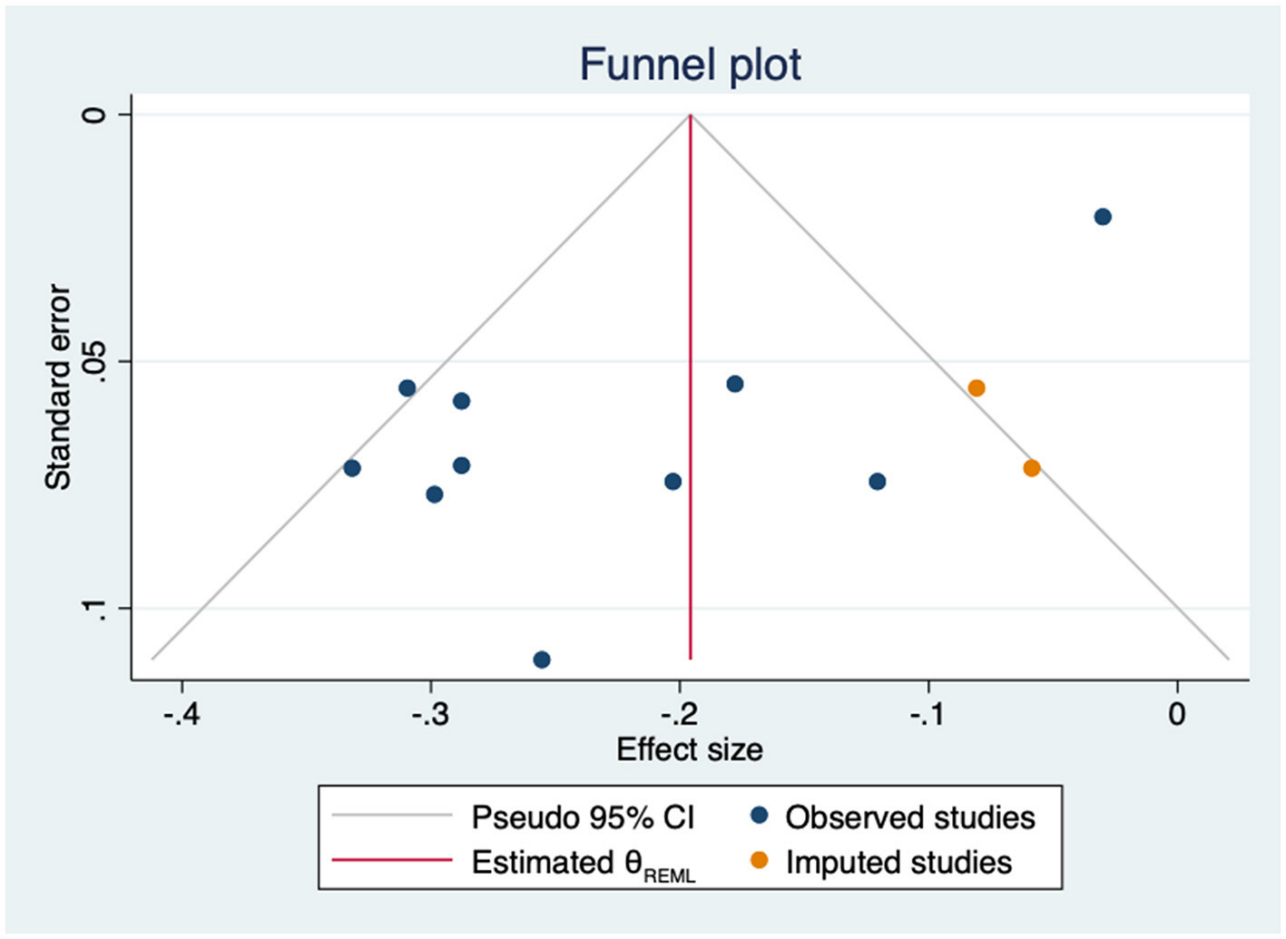
Funnel plot with trim-and-fill imputations for the association between empowerment and anxiety.

Sensitivity analysis using the leave-one-out approach identified one outlier study (36). When it was excluded, the heterogeneity decreased to 14.33% (Q = 8.72, *p* = 0.366) and the effect increased to −0.25 (95% CI from −0.29 to −0.20) (Supplementary material 4A). In the univariate meta-regression analyses, nor age (β = 0.00; 95%CI −0.01 to 0.01; k = 9); gender (β = 0.00; 95%CI −0.00 to 0.01; k = 9); HbA1C level (β = 0.08; 95%CI −0.01 to 0.16; k = 6) nor years since diagnosis (β = 0.02; 95%CI −0.01 to 0.05; k = 7) obtained significant results. The regression-based Egger's test showed evidence of small-study effects (*p* = 0.02) and the funnel-plot analysis was asymmetrical, indicating publication bias. Trim-and-fill analysis by the imputation of two studies on the right side resulted in a lower, but still statistically significant correlation coefficient (*r* = −0.20, 95%CI −0.26 to −0.13) (Figure 4).

### 3.3. The association between empowerment-related constructs and depression

The relationship between empowerment-related constructs and depressive symptoms was reported in thirty-five studies (*n* = 9315) (34–36, 47, 48, 54, 55, 58, 61–63, 65, 66, 69, 71, 73, 75, 77, 78, 83, 84, 86–90, 92, 93, 96, 105–108, 110, 111). The most frequently used empowerment construct was self-efficacy (*n* = 27) (35, 54, 55, 58, 61–63, 65, 66, 69, 71, 73, 75, 77, 78, 80, 83, 84, 86–90, 92, 93, 96, 105, 106), followed by patient empowerment itself (*n* = 4) (34, 36, 47, 48), perceived control (*n* = 2) (110, 111) and patient activation (*n* = 2) (107, 108). Two studies were not included in the quantitative analysis due the lack of numerical data (65, 83) and Sacco et al. (92) was also excluded because its sample overlapped with Sacco et al. (93). Matteucci et al. (83) found a negative association between self-efficacy and depression, whereas in the study of Coffman et al. (65) the association between diabetes self-efficacy and depression was not significant.

**Figure 5:**
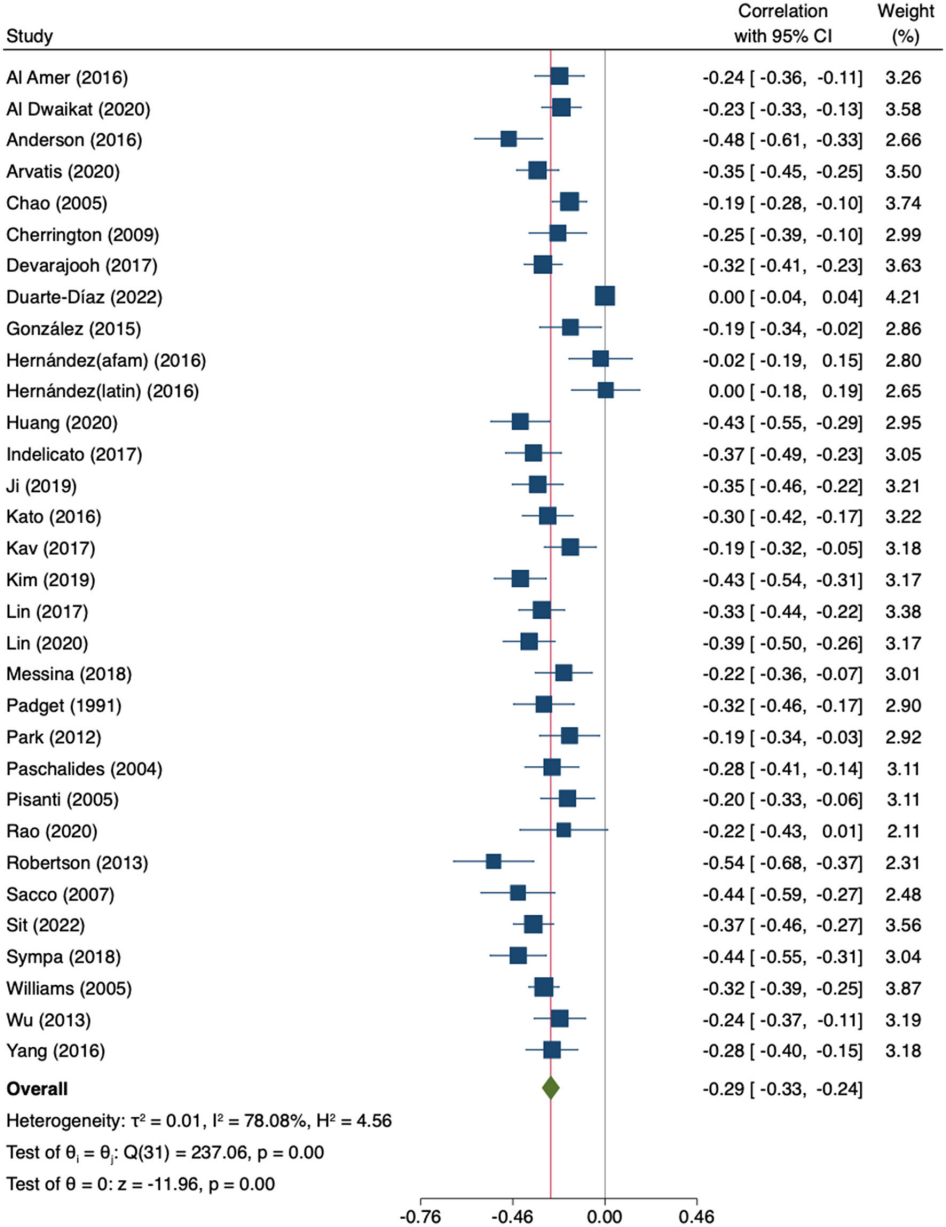
Random-effects meta-analysis on the association between empowerment and depression.

Through a MA including thirty correlation coefficients from twenty-nine original studies (*n* = 8,990) (34–36, 47, 48, 54, 55, 58, 61–63, 66, 69, 71, 73, 75, 77, 78, 80, 84, 86–90, 93, 96, 105–108, 110, 111), a weak-to-moderate inverse correlation was found (*r* = −0.29; 95%CI −0.33 to −0.24; *I*^2^ = 78.08%; k = 32) (Figure 5).

**Figure 6:**
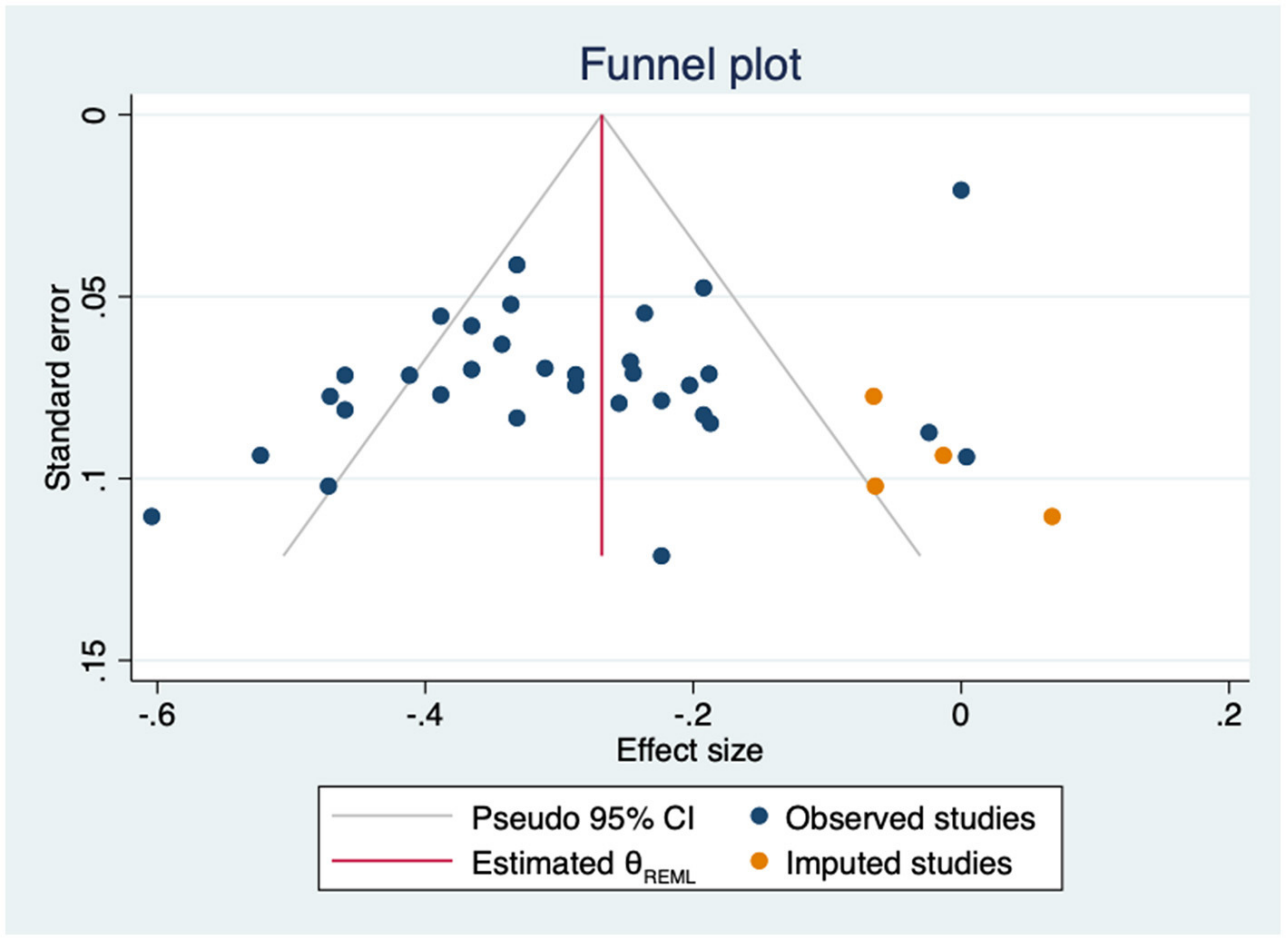
Funnel plot with trim-and-fill imputations for the association between empowerment and depression.

The leave-one-out MA showed that neither the direction nor significance of the pooled effect changed after the removal of any individual study (Supplementary material 4B). The estimates ranged from −0.28 to −0.30. There was significant heterogeneity across studies (Q = 237.06; *p* < 0.000). The subgroup analysis by empowerment-related construct showed no significant differences (*p* = 0.455), not even when comparing specific empowerment measures against the other combined empowerment-related constructs (*p* = 0.143) (Supplementary material 5A). However, in in the subgroup of studies using patient empowerment scales, heterogeneity was high and the association with depressive symptoms was not statistically significant. In the univariate meta-regression analyses, both age and female gender significantly moderated the association between patient empowerment-related constructs and depression (age: β = −0.01; 95%CI −0.02 to −0.00; k = 31; gender: β = 0.00; 95%CI 0.00 to 0.01; k = 31). Age accounted for 17% of heterogeneity and gender for 18%. In the multivariate analysis, only gender remained significant (β = 0.00; 95%CI 0.00 to 0.01; k = 30). Neither HbA1c levels (β = −0.07; 95%CI −0.17 to 0.03; k = 17) nor years since diagnosis (β = −0.00; 95%CI −0.03 to 0.02; k = 22) were found to be significant moderators. The regression-based Egger's test was not significant (*p* = 0.08), however Duval and Tweedie's trim-and-fill analysis suggested that four studies were missing on the right side of the mean effect (Figure 6). The imputation of these four studies did not substantially change the result (*r* = −0.27; 95%CI −0.32 to −0.22, k = 36).

### 3.4. The association between empowerment-related constructs and distress

Twenty studies (*n* = 7,396) (35, 48, 50, 51, 53, 55–58, 60, 64, 66, 67, 69, 71, 76, 78, 84, 90, 106) reported the association between empowerment-related constructs and general or diabetes-related distress. Most of the studies used self-efficacy scales (*n* = 17) (35, 53, 55–58, 60, 64, 66, 67, 69, 71, 76, 78, 84, 90, 106) whereas a specific scale addressing diabetes patient empowerment was used in three studies (48, 50, 51). One study, not included in the MA due to the lack of numerical data (57), reported that distress levels were significantly lower among those T2DM patients who reported higher self-efficacy.

**Figure 7:**
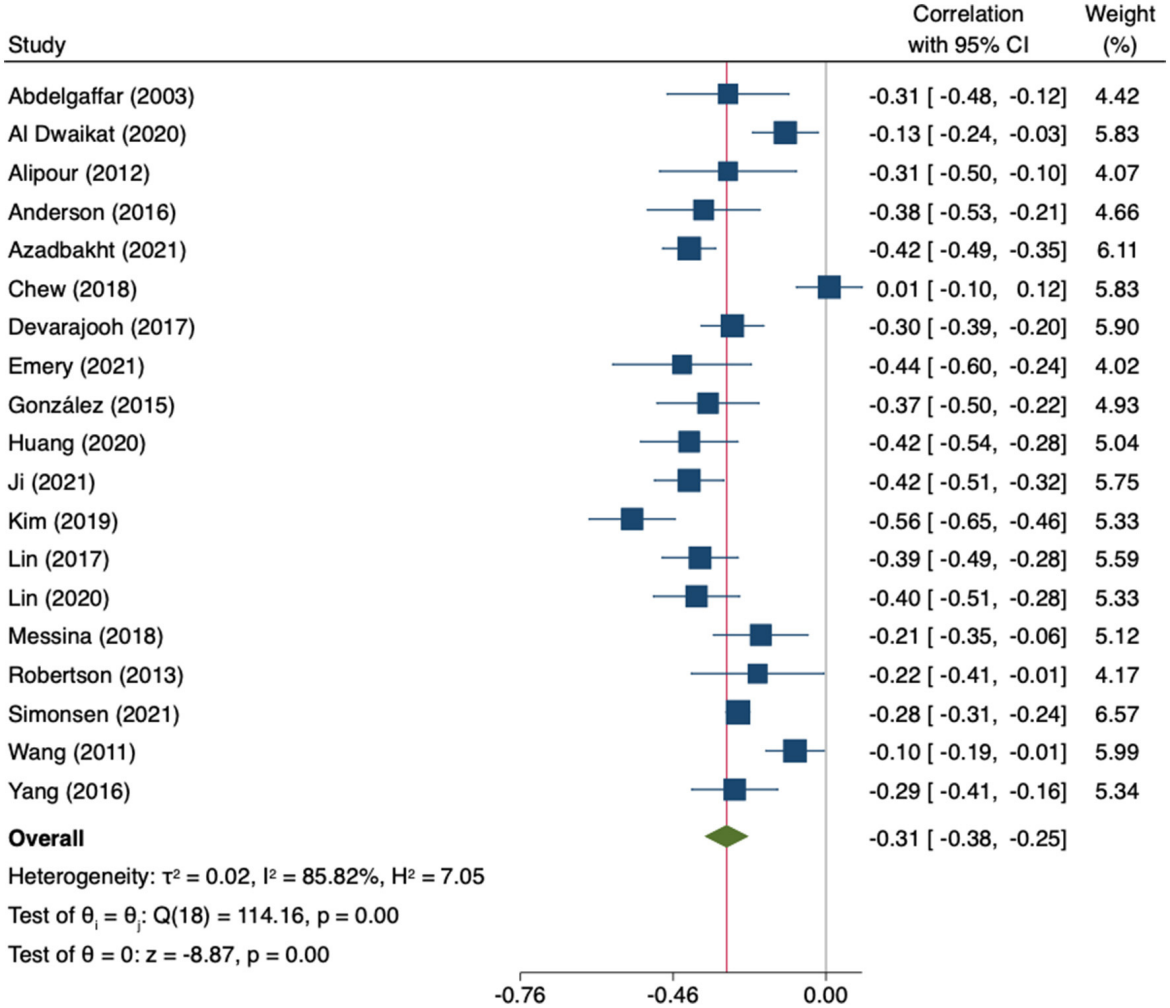
Random-effects meta-analysis on the association between empowerment and distress.

The MA of the remaining nineteen studies (*n* = 6,700) showed a moderate inverse correlation between empowerment-related constructs and distress (r = −0.31; 95%CI −0.38 to −0.25; *I*^2^ = 86%; k = 19) (Figure 7).

**Figure 8:**
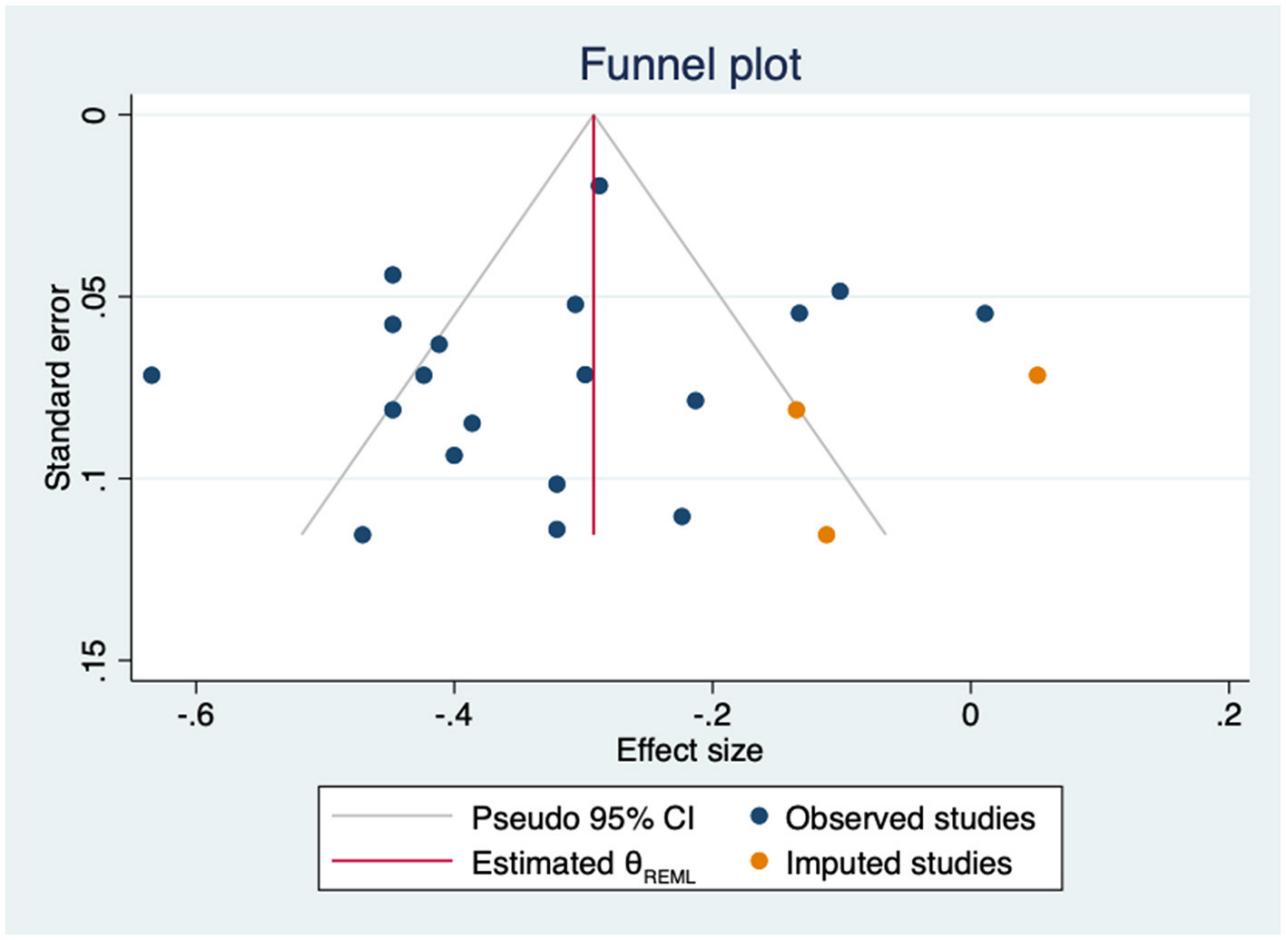
Funnel plot with trim-and-fill imputations for the association between empowerment and distress.

The leave-one-out analysis showed no relevant influence of any individual study. The estimates ranged from −0.30 to −0.33 (Supplementary material 4C). Heterogeneity was high and statistically significant (Q = 114.16, *p* = 0.000). In the subgroup analysis by type of empowerment construct, no statistically significant differences were found (*p* = 0.439). The correlation was stronger in studies using specific diabetes-related distress measures than in studies using general ones (*p* = 0.03) (Supplementary material 5B). Likewise, none of the sociodemographic or clinical variables significantly modified the association between empowerment-related constructs and distress (age: β = 0.01; 95%CI −0.01 to 0.01; k = 18; gender: β = 0.00; 95%CI −0.00 to 0.00; k = 18; HbA1c: β = 0.09; 95%CI −0.11 to 0.25; k = 8; and years since diagnosis: β = −0.02; 95%CI −0.05 to 0.01; k = 14). No evidence of small-study effects was identified by the Egger test (*p* = 0.348). Nevertheless, the imputation of three coefficients in the right side of the plot (Figure 8) slightly decreased the association (*r* = −0.29, 95%CI −0.37 to −0.22, k = 22).

### 3.5. The association between empowerment-related constructs and quality of life

#### 3.5.1. General quality of life

Twenty-two studies (*n* = 5,005) (44–46, 49, 51, 52, 56, 68, 70, 72, 74, 79, 81, 82, 84, 85, 91, 95, 98–100, 104) evaluated the relationship between empowerment-related constructs and general QoL in patients with T2DM. Specific patient empowerment scales were used in six studies (44–46, 49, 51, 52) while self-efficacy was used in the remaining sixteen studies (56, 68, 70, 72, 74, 79, 81, 82, 84, 85, 91, 95, 98–100, 104). One study (44) used ANOVA to quantify this association and was not included in the MA. The aim of this study was to identify predictors of good QoL among diabetic patients in Ghana and Nigeria, reporting that patient empowerment only significantly predicted QoL in Nigeria.

**Figure 9:**
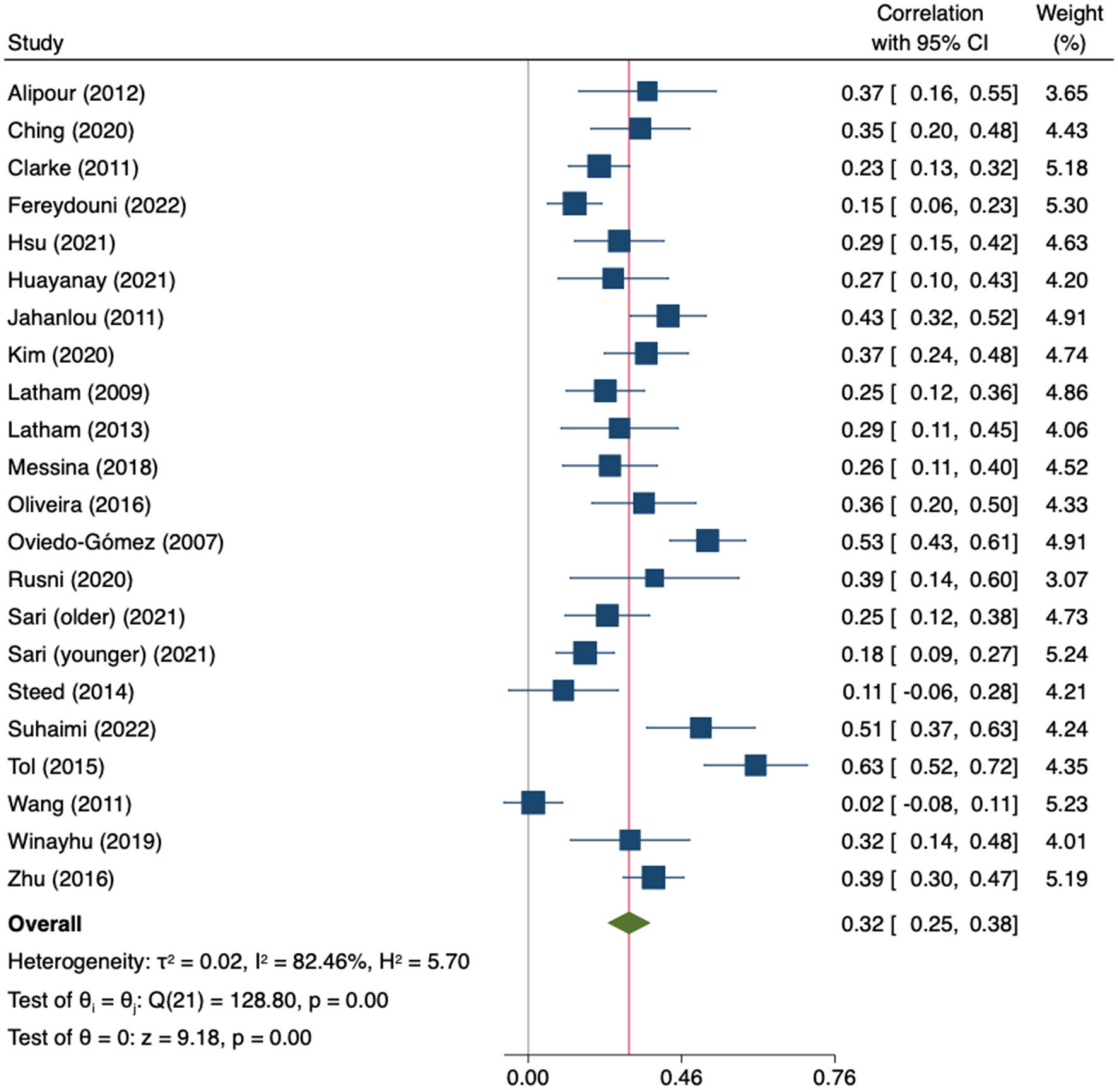
Random-effects meta-analysis on the association between empowerment and general QoL.

Twenty-two correlation coefficients from twenty-one studies (*n* = 4,609) were pooled. A moderate positive correlation was found but heterogeneity across studies was high (*r* = 0.32; 95%CI 0.25–0.38; *I*^2^ = 82.46%, k = 22) (Figure 9).

**Figure 10:**
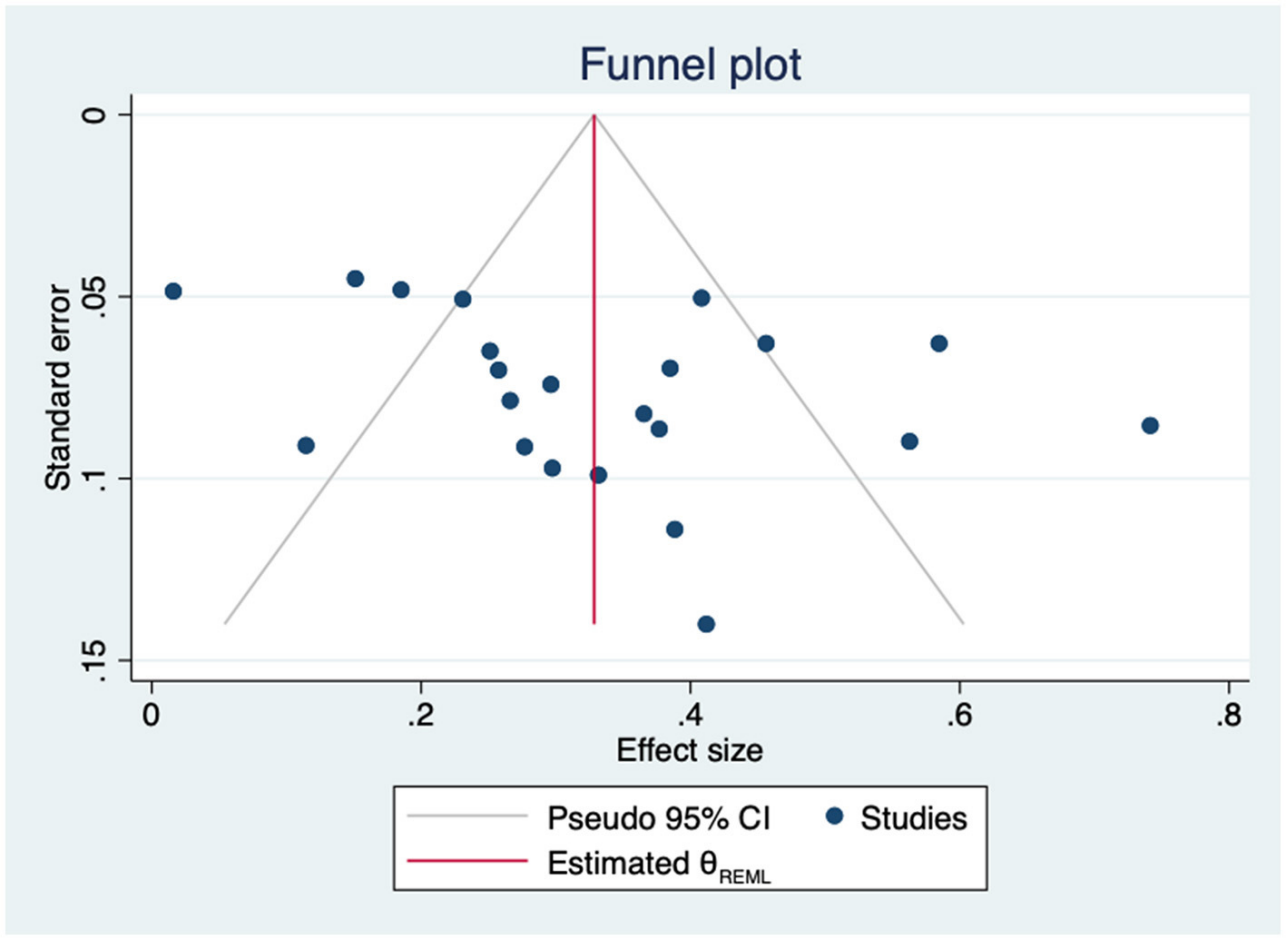
Funnel plot for the association between empowerment and general QoL.

The leave-one-out sensitivity analysis did not identify any study whose exclusion substantially modified the result. The estimates ranged from 0.30 to 0.33 (Supplementary material 4D). Heterogeneity across studies was high and statistically significant (Q = 128.80, *p* = 0.000). The subgroup analyses by construct (empowerment vs. self-efficacy) and by type of study (cross-sectional vs. longitudinal) did not find statistically significant differences (Supplementary material 5C). Other sources of heterogeneity were analyzed through meta-regression, but neither age (β = −0.00; 95%CI −0.01 to 0.01; k = 19), gender (β = 0.00; 95%CI −0.00 to 0.00; k = 20), HbA1c (β = 0.04; 95%CI −0.17 to 0.24; k = 6), nor years since diagnosis (β = −0.02; 95%CI −0.06 to 0.02; k = 11) found significant results. The Egger tests showed no evidence of small-study effects (*p* = 0.121) and the trim-and-fill model suggested that no imputation or adjustment was needed (Figure 10).

#### 3.5.2. Mental quality of life

**Figure 11:**
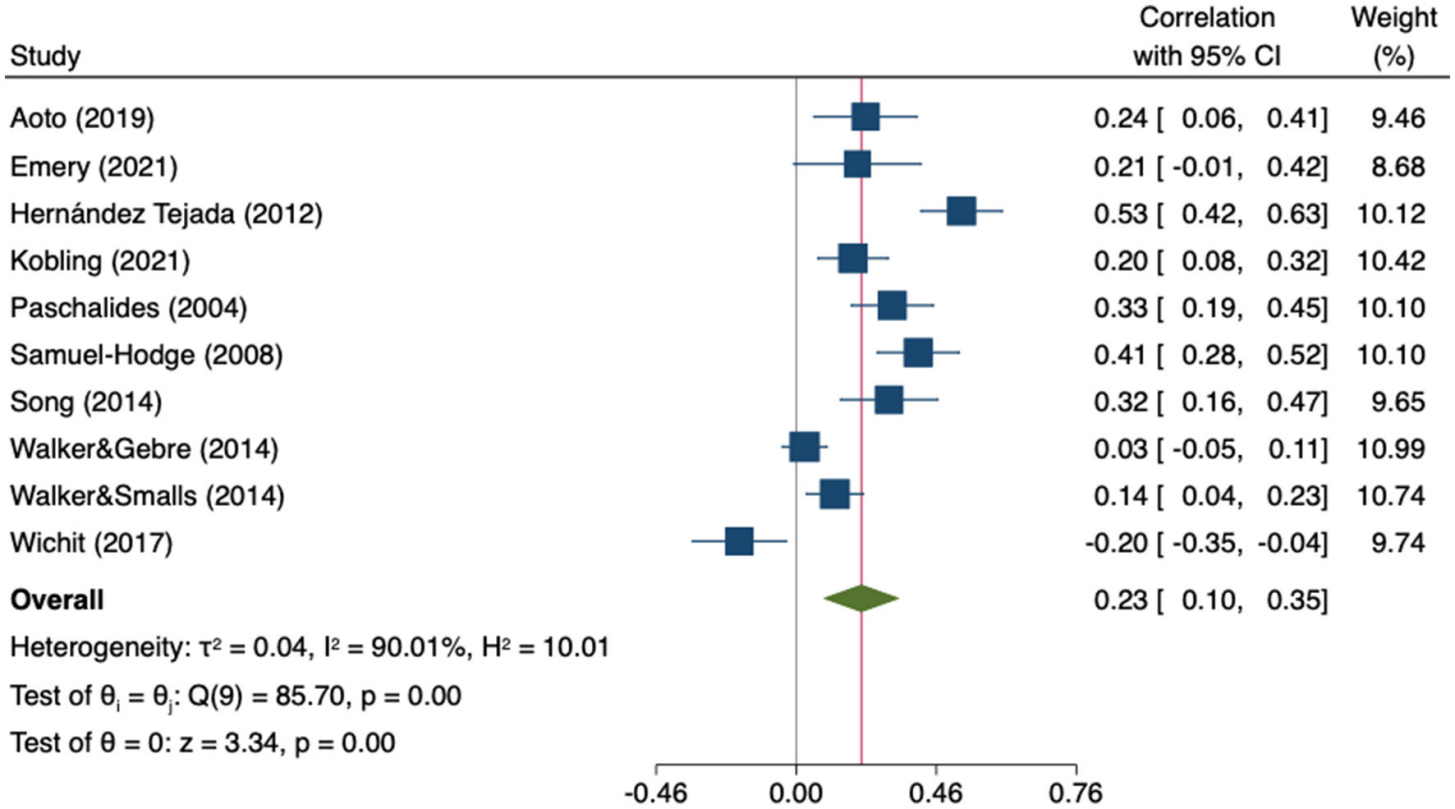
Random-effects meta-analysis on the association between empowerment and mental QoL.

Eight studies (*n* = 2,267) reported data on the association between self-efficacy and the mental component of QoL (59, 67, 80, 94, 97, 101–103) and two did so with perceived control (109, 110). The MA of these ten studies showed a significant positive correlation (*r* = 0.23; 95%CI 0.10 to 0.35; *I*^2^ = 90%; k = 10) (Figure 11).

**Figure 12:**
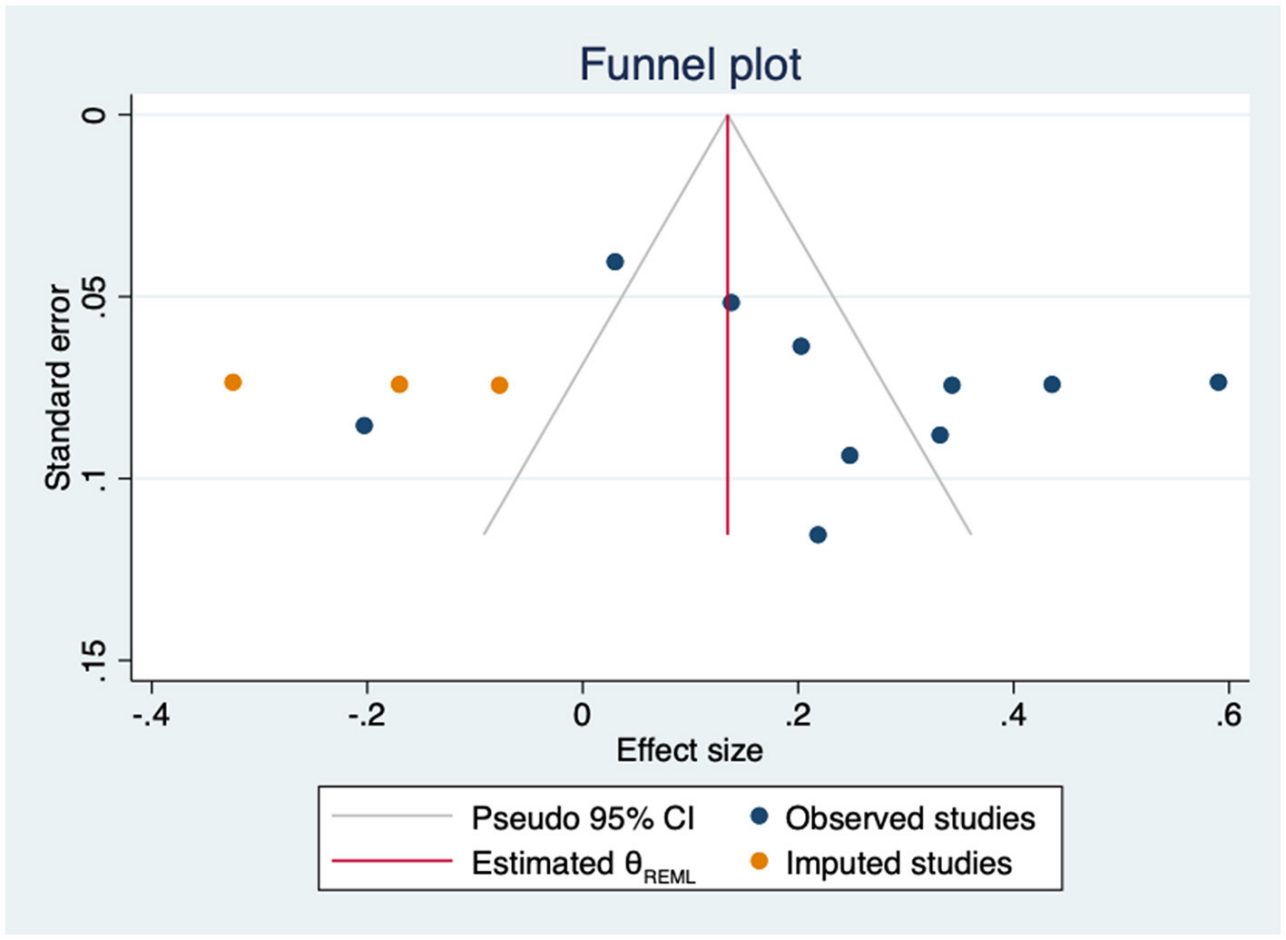
Funnel plot with trim-and-fill imputations for the association between empowerment and mental QoL.

The sensitivity analysis showed that neither the direction nor significance of the pooled effect changed after removing any individual study. The estimates ranged from 0.19 to 0.27 (Supplementary material 4E). Heterogeneity was high and statistically significant (Q = 85.70, *p* = 0.000). Subgroup analysis revealed that the correlation coefficient in studies using perceived control scales was significantly higher than in those using self-efficacy scales (*p* = 0.037) (Supplementary material 5D). The moderator analyses through meta-regression showed no statistical effect of age (β = 0.03; 95%CI −0.04 to 0.10; k = 8), gender (β = −0.00; 95%CI −0.02 to 0.02; k = 10), HbA1c (β = 0.21; 95%CI −0.04 to 0.46; k = 7) or years since diagnosis (β = 0.05; 95%CI −0.02 to 0.11; k = 6). The Egger tests showed no evidence of small-study effects (*p* = 0.675), however, Duval and Tweedie's trim-and-fill analysis suggested that three studies were missing on the left side of the mean effect (Figure 12). When these three studies were imputed, the association was no longer statistically significant (*r* = 0.13; 95%CI −0.01 to 0.28, k = 13).

#### 3.5.3. Physical quality of life

**Figure 13:**
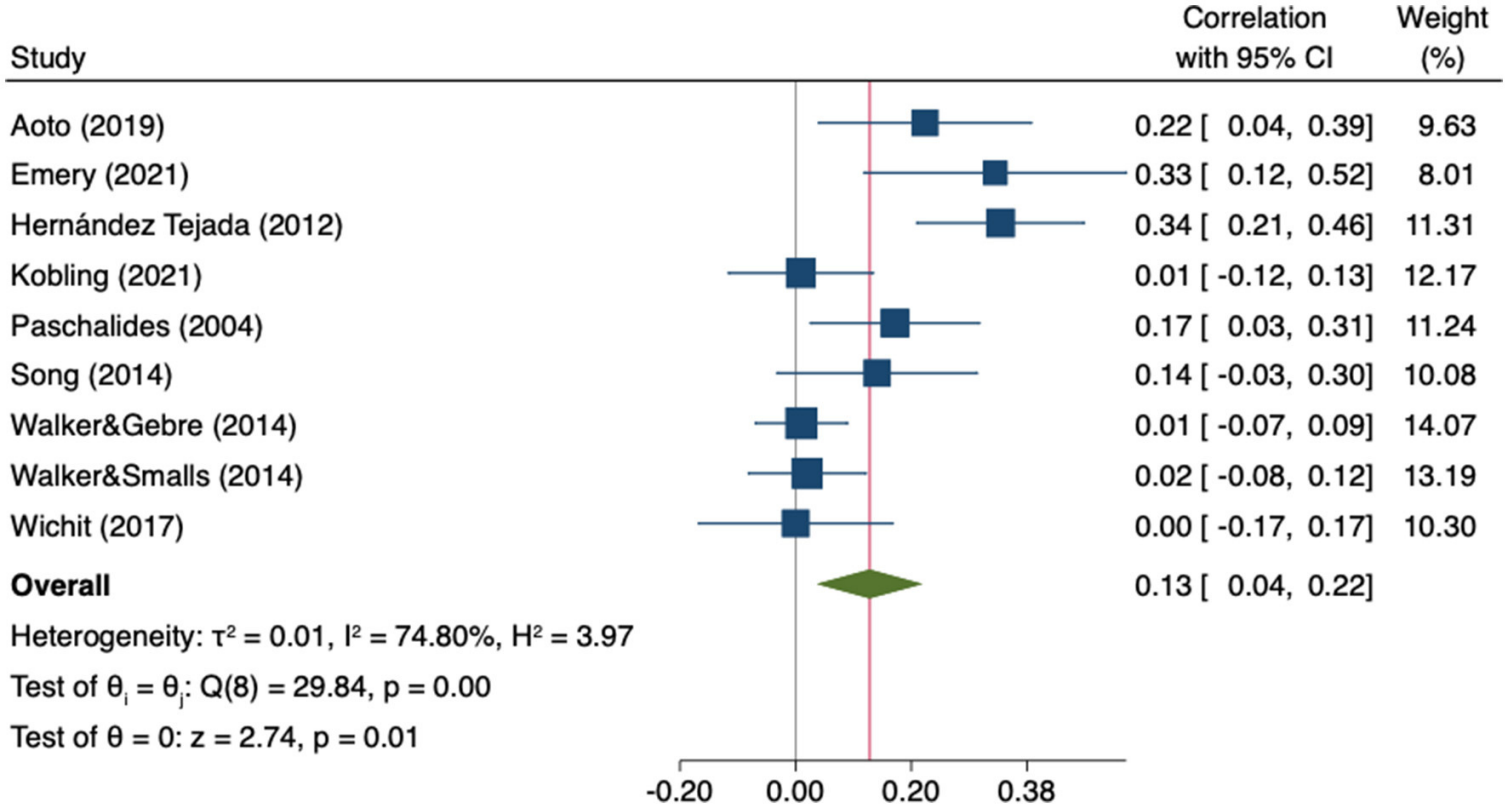
Random-effects meta-analysis on the association between empowerment and physical QoL.

Seven studies (*n* = 2,082) reported data on the association between self-efficacy and the physical component of QoL (59, 67, 80, 97, 101–103) and two did so with perceived control (109, 110). A weak positive significant correlation with physical QoL was observed (r = 0.13; 95%CI 0.04 to 0.22; *I*^2^ = 74.80%, k = 9) in the MA of the nine studies (Figure 13).

**Figure 14:**
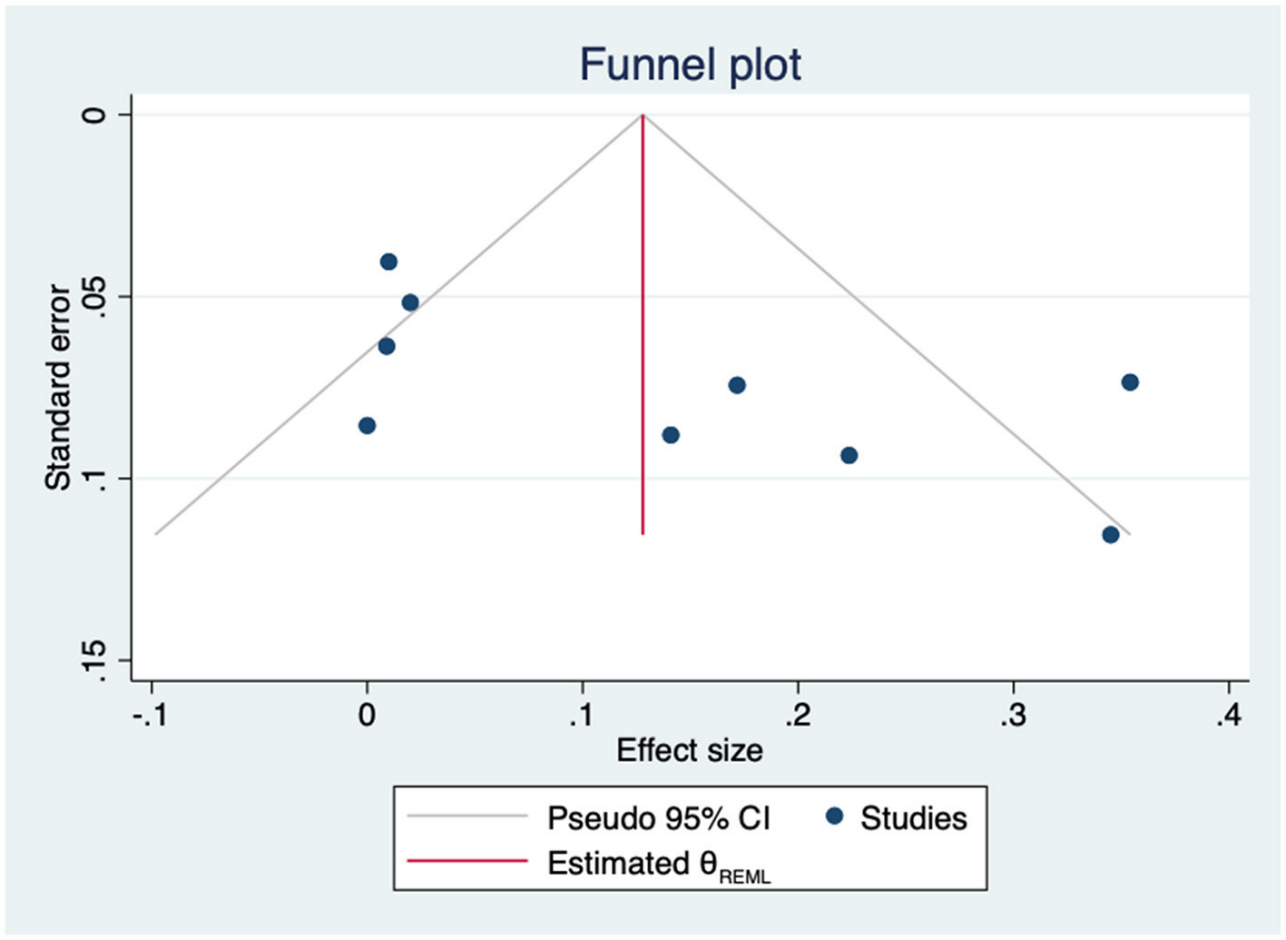
Funnel plot for the association between empowerment and physical QoL.

The sensitivity analysis showed the consistency of the findings as neither direction or significance of the pooled effect changed after removing any individual study. The estimates ranged from 0.09 to 0.14 (Supplementary material 4F). Heterogeneity was high and statistically significant (Q = 29.84, *p* = 0.0002). No statistically significant differences were found in the subgroup analysis by type of empowerment indicator (self-efficacy vs. perceived control) (Supplementary material 5E). The visual inspection of the funnel plot suggested showed asymmetry (Figure 14).

## 4. Discussion

To the best of our knowledge, this SR is the first synthesis of the available knowledge on the relationship between patient empowerment or related constructs with affective outcomes and QoL in patients with T2DM. Seventy-one studies were identified, including a large total sample of adults with T2DM. However, only a few studies have evaluated empowerment itself, with most of them relying on self-efficacy measures, since this construct has a longer tradition in psychology than patient empowerment. As a result, the results mainly refer to this specific component of empowerment, i.e., the self-perception of being able to execute behaviors necessary to resolve specific problems or tasks (the self-management of the disease in this case). As mentioned in the introduction, empowerment has a broader theoretical scope than constructs such as self-efficacy or perceived control. For instance, it includes not only the subjective perception of self-efficacy, but also the objective cognitive and emotional abilities required for adequate disease management (e.g., correct knowledge of self-care strategies, objective health literacy, adaptive coping styles or good communication skills when interacting with healthcare providers). However, the different theoretical models of empowerment agree that self-efficacy is one of its essential components, and it is not conceivable to have an empowered patient with a poor perception of their ability to correctly manage their health condition correctly. There is empirical evidence showing that empowerment and self-efficacy are different and not interchangeable concepts, but they are also significantly correlated (125–127). Our subgroup analyses based on the evaluated construct were inconclusive due to the small number of studies assessing empowerment. Nevertheless, the pooled results of these studies were also significant in the same direction as the association between self-efficacy and the outcomes, albeit the strength of the association may be weaker, especially for depression.

The results of the MA showed significant inverse relationships between these constructs and anxiety, depression and distress. Besides, there are significant positive associations with mental, physical and general QoL Nevertheless, the number of studies/patients is lower for the mental and physical components, as well as the strength of their correlations with the empowerment-related constructs. The effect sizes are small, between 0.13 and 0.32 in absolute values. Results on depression showed a significant moderator effect of gender. Specifically, when the percentage of females in the samples increased, so did the observed inverse correlation between empowerment and depressive symptoms (nonetheless, a significant proportion of the variance remained unexplained after controlling for gender). We do not know of other studies in diabetes that have analyzed this moderator effect of gender. In a study with family caregivers of elderly people, a lower self-efficacy was associated with depressive symptoms and this relationship was stronger in female caregivers (128).

Although publication bias cannot be ruled out, the pooled correlations did not change substantially and remained significant when potential missed studies were imputed. The main uncertainty relating to these results has to do with the high heterogeneity observed in all the analyses, which was mostly unexplained by the moderators studied (except by one outlier study causing most of the heterogeneity in the case of anxiety). The few subgroup analyses conducted with the available data are inconclusive because all of them are limited by the low number of studies in one of the subgroups. Another methodological limitation of the identified evidence is that most studies were cross-sectional, ruling out the possibility of investigating longitudinal associations. Only four prospective studies reported this association for QoL and could be included in a subgroup analysis, showing a slightly lower correlation than that observed in cross-sectional studies (0.25 vs. 0.33), although this difference was not significant. Duarte-Díaz et al. (36) found that baseline empowerment was not a significant predictor of anxiety and depression 1 and 2 years later, but the change in empowerment significantly correlated with an inverse change in affective outcomes. Future studies should try to overcome these limitations, including prospective designs, interaction analyses with gender or other sociodemographic or clinical variables, and also comparing the predictive capacity of the different empowerment-related constructs on glycemic control and acute complications.

The American Psychological Association defines psychological distress as “*a set of painful mental and physical symptoms that are associated with normal fluctuations of mood in most people*” (129). Likewise, diabetes-related distress implies significant negative emotional reactions to the diagnosis of diabetes, the threat of complications and self-management demands (130). According to the results here, lower levels of empowerment-related constructs, especially self-efficacy, are linked to both general and diabetes-related distress, but the strength of the association was higher with specific diabetes distress scales. Although this result is provisional due to the low number of studies in the subgroup of general distress, it seems reasonable to expect that patient empowerment, which includes knowledge, skills, attitudes and self-awareness to influence one's own health, is mainly related to the specific affective processes arising due to the difficulty of managing the demands of diabetes. The positive impact of empowerment and related constructs on psychological symptoms and distress results in a better mental and general quality of life; earlier SRs among patients with other health conditions such as cardiovascular disease (131), cancer (132) and rheumatoid arthritis (133) have shown significant positive associations between empowerment-related constructs and quality of life.

The correlation between self-efficacy about diabetes self-care and affective outcomes aligns with Bandura's postulates (134). Self-efficacy plays a pivotal role in regulating affective states (135). Patients with lower self-efficacy may harbor doubts about their abilities to cope with the daily demands of diabetes, resulting in increased stress, anxiety or depression. On the other side, the possibility of bidirectional associations cannot be ruled out and the patients' ability and willingness to be actively involved in their care may be affected by the presence of affective symptoms. Depressed patients may feel that they have less control over the disease, thus resulting in poorer self-care strategies (136). Likewise, anxiety can interfere on cognitive and motivational processes necessary for an adequate self-care and the emergence of empowerment (137). On the contrary, empowering patients may improve affective symptoms. A recent MA showed that interventions tailoring patient activation effectively improve anxiety and depression symptoms in several chronic conditions including diabetes (138). Similarly, Hernández-Jimenez et al. (39), found that a 2-years comprehensive program based on empowerment strategies had a noteworthy positive effect on both anxiety and depression symptoms in recently diagnosed T2DM patients.

The findings here have some relevant implications for clinical practice. Previous studies have emphasized the importance of managing emotional symptoms in patients with T2DM and that empowerment-related constructs might play a significant role. The association between affective symptoms and diabetes self-care and treatment adherence has been widely studied but what the influence of these pathways is has still not been fully explained. Affective symptoms are related to lower self-efficacy and low self-efficacy is equally associated with poor glycemic control and lower medication adherence (139). Moreover, several studies highlight empowerment-related constructs as potential mediators between affective outcomes and self-care and diabetes control. Specifically, there is evidence suggesting that both depression and diabetes distress are related to poorer treatment adherence, self-care behaviors and glycemic control while this association is partially mediated through perceived control or self-efficacy (48, 66, 69). In addition, a recent study including patients with type 1 DM and T2DM has shown that the association between self-efficacy and QoL was partially mediated by depressive symptoms (140). Accordingly, it is necessary to establish how these variables are related to each other to better understand the pathways through which patient empowerment is related to affective symptoms and QoL and how they jointly affect self-management and glycemic control in patients with T2DM.

The main methodological limitation of this SR is the possibility of missing studies not included in the databases used. Furthermore, gray literature was not included in the search strategy and this may have resulted in a loss of information. Other limitations concern to the identified studies and have been previously commented. Most of them are cross-sectional, precluding conclusions about longitudinal associations. A high statistically significant heterogeneity was obtained in all the analyses, mostly unexplained by the studied moderators. Subgroup analyses were limited by the low number of studies in one of the subgroups, and there could be non-considered confounding variables that potentially moderated the observed associations. Finally, despite the fact that publication bias was not identified, the Egger tests may lack the statistical power to detect bias due to the small number of included studies in the case of anxiety and both mental and physical QoL.

## 5. Conclusion

Current evidence suggests that empowerment-related constructs are negatively associated with affective symptoms and positively correlated with QoL in patients with T2DM. A wide range of variables can affect psychological outcomes and thus these associations are complex. In accordance, the correlation coefficients we reported are mostly small but not negligible. This evidence is mainly from cross-sectional studies and thus it is not possible to confirm the direction of the observed association. Consequently, high-quality prospective studies are warranted not only to better understand the role of patient empowerment and other indicators on affective symptoms and QoL but also to assess causal associations. Moreover, variables potentially modifying the association between empowerment-related constructs and affective outcomes and QoL remain unclear and require further investigation.

### 5.1. Practice implications

The findings of this SR provide valuable information to researchers, healthcare professionals and policy makers involved in the management of T2DM. The results highlight patient empowerment and related constructs as significant components of diabetes care linked to better mental health and increased QoL. Thus, this should be considered in the design, development and implementation of effective interventions and policies that seek to improve clinical and psychosocial outcomes in patients with T2DM.

## Data availability statement

The original contributions presented in the study are included in the article/Supplementary material, further inquiries can be directed to the corresponding author.

## Author contributions

AD-D: conceptualization, methodology, formal analysis, investigation, and writing—original draft. LP-P: conceptualization, methodology, supervision, and writing—review and editing. AR-S: conceptualization, methodology, formal analysis, and writing—review and editing. WP: conceptualization, supervision, and writing—review and editing. YÁ-P, VR-G, LG-A, MB-B, and SG-M: investigation and writing—review and editing. HG-P, YR-F, and CC: writing—review and editing. PS-A: supervision and writing—review and editing. All authors contributed to the article and approved the submitted version.
