# Supplementary material for: The relationship between patient empowerment and related constructs, affective symptoms and quality of life in patients with type 2 diabetes: a systematic review and meta-analysis
Source: Front Public Health. 2023 Apr 17;11:1118324. doi: 10.3389/fpubh.2023.1118324 (PMC10150112; doi:10.3389/fpubh.2023.1118324)
Supplement: Supplementary file 2 [file Data_Sheet_2.docx]

Supplementary Material 2

The Relationship Between Patient Empowerment and Related Constructs, Affective Symptoms and Quality of Life in Patients with Type 2 Diabetes: A Systematic Review and Meta-Analysis

Andrea Duarte-Díaz^1,2,3,4^, Lilisbeth Perestelo-Pérez^3,4,5^*, Amado Rivero-Santana^1,3,4^, Wenceslao Peñate^2^, Yolanda Álvarez-Pérez^1,3,4^, Vanesa Ramos-García^1,3,4^, Himar González-Pacheco^1,3,4^, Libertad Goya-Arteaga^6^, Miriam de Bonis-Braun^6^, Silvia González-Martín^6^, Yolanda Ramallo-Fariña^1,3,4^, Carme Carrion^3,7^ and Pedro Serrano-Aguilar^3,4,5^

*** Correspondence:** Lilisbeth Perestelo-Pérez: lilisbeth.presteloperez@sescs.es

# Supplementary Material 2. Search strategy

| **Ovid MEDLINE** | | |
| --- | --- | --- |
| **#** | **Searches** | **Results** |
| Ovid MEDLINE(R) ALL <1946 to July 18, 2022> | | |
| 1 | Diabetes Mellitus, Type 2/ | 159452 |
| 2 | (non insulin* depend* or noninsulin* depend* or noninsulin?depend* or non insulin?depend*).tw. | 12398 |
| 3 | ((typ? 2 or typ? II or typ?2 or typ?II) adj3 diabet*).tw. | 172607 |
| 4 | (MODY or NIDDM or T2D*).tw. | 50178 |
| 5 | (((late or adult* or matur* or slow or stabl*) adj3 onset) and diabet*).tw. | 5133 |
| 6 | 1 or 2 or 3 or 4 or 5 | 227849 |
| 7 | exp Diabetes Insipidus/ | 8220 |
| 8 | diabet* insipidus.tw. | 9201 |
| 9 | 7 or 8 | 11501 |
| 10 | 6 not 9 | 227721 |
| 11 | ((patient$ or adult$ or client$ or participant$ or individual$) adj3 empower$).tw. | 6245 |
| 12 | Self Efficacy/ or (self efficacy or self-efficacy).tw. | 43918 |
| 13 | patient activation.mp. or Patient Participation/ | 29616 |
| 14 | Empowerment/ | 684 |
| 15 | 11 or 12 or 13 or 14 | 78339 |
| 16 | 6 and 15 | 1912 |
| 17 | Anxiety/ or Anxiety Disorders/ or anxiety.tw. | 266633 |
| 18 | depression.mp. or Depression/ or Depressive disorders/ or depressive disorder*.mp. | 462584 |
| 19 | "Quality of Life"/ or quality of life.tw. | 415137 |
| 20 | 17 or 18 or 19 | 972125 |
| 21 | 16 and 20 | 438 |

| **EMBASE** |
| --- |
| **Results** |
| **1,157** |
| **#28** |
| **#19** AND **#27** |
| **1,322,900** |
| **#27** |
| **#20** OR **#21** OR **#22** OR **#23** OR **#24** OR **#25** OR **#26** |
| **418,653** |
| **#26** |
| **'quality of life'**:ti,ab |
| **486,285** |
| **#25** |
| **'quality of life'**/exp |
| **40,851** |
| **#24** |
| ((**depression** OR **depressive**) NEAR/1 **disorder**):ti,ab |
| **495,367** |
| **#23** |
| **'depression'**/exp |
| **271,311** |
| **#22** |
| **anxiety**:ti,ab |
| **247,443** |
| **#21** |
| **'anxiety disorder'**/exp |
| **213,637** |
| **#20** |
| **'anxiety'**/exp |
| **3,232** |
| **#19** |
| **#10** AND **#18** |
| **252,639** |
| **#18** |
| **#11** OR **#12** OR **#13** OR **#14** OR **#15** OR **#16** OR **#17** |
| **9,127** |
| **#17** |
| **'empowerment'**/exp |
| **5,86** |
| **#16** |
| (**patient*** NEAR/1 (**activation** OR **participation**)):ti,ab |
| **48** |
| **#15** |
| **'patient activation'**/exp |
| **27,085** |
| **#14** |
| **'patient participation'**/exp |
| **38,174** |
| **#13** |
| **'self concept'**:ti,ab OR **'self efficacy'**:ti,ab OR **'self-efficacy'**:ti,ab |
| **199,671** |
| **#12** |
| **'self concept'**/exp |
| **7,555** |
| **#11** |
| ((**patient*** OR **adult*** OR **client*** OR **participant*** OR **individual***) NEAR/3 **empower***):ti,ab |
| **311,158** |
| **#10** |
| **#6** NOT **#9** |
| **16,512** |
| **#9** |
| **#7** OR **#8** |
| **11,058** |
| **#8** |
| **'diabet* insipidus'**:ti,ab |
| **15,079** |
| **#7** |
| **'diabetes insipidus'**/exp |
| **311,513** |
| **#6** |
| **#1** OR **#2** OR **#3** OR **#4** OR **#5** |
| **6,782** |
| **#5** |
| (((**late** OR **adult*** OR **matur*** OR **slow** OR **stabl***) NEAR/3 **onset**):ti,ab) AND **diabet***:ti,ab |
| **65,071** |
| **#4** |
| **mody**:ti,ab OR **niddm**:ti,ab OR **t2d***:ti,ab |
| **221,759** |
| **#3** |
| ((**'typ? 2'** OR **'typ? ii'** OR **'typ?2'** OR **'typ?ii'**) NEAR/3 **diabet***):ti,ab |
| **14,287** |
| **#2** |
| **'non insulin* depend*'**:ti,ab OR **'noninsulin* depend*'**:ti,ab OR **'noninsulin?depend*'**:ti,ab OR **'non insulin?depend*'**:ti,ab |
| **256,585** |
| **#1** |
| **'non insulin dependent diabetes mellitus'**/exp |

| **Cochrane Library** | | |
| --- | --- | --- |
| ID | Search | Hits |
| #1 | MeSH descriptor: [Diabetes Mellitus, Type 2] explode all trees | 20070 |
| #2 | (non insulin* depend* or noninsulin* depend* or noninsulin?depend* or non insulin?depend*):ti,ab | 3011 |
| #3 | ((typ? 2 or typ? II or typ?2 or typ?II) NEAR/3 diabet*) | 57649 |
| #4 | (MODY or NIDDM or T2D*):ti,ab | 12148 |
| #5 | (((late or adult* or matur* or slow or stabl*) NEAR/3 onset) and diabet*) | 447 |
| #6 | #1 or #2 or #3 or #4 or #5 | 59666 |
| #7 | MeSH descriptor: [Diabetes Insipidus] explode all trees | 71 |
| #8 | diabet* insipidus:ti,ab | 129 |
| #9 | #7 or #8 | 153 |
| #10 | #6 not #9 | 59659 |
| #11 | ((patient* or adult* or client* or participant* or individual*) NEAR/3 empower*) | 1361 |
| #12 | MeSH descriptor: [Self Efficacy] explode all trees | 3465 |
| #13 | (self efficacy or self-efficacy):ti,ab | 31744 |
| #14 | MeSH descriptor: [Patient Participation] explode all trees | 1524 |
| #15 | patient activation:ti,ab | 6515 |
| #16 | MeSH descriptor: [Empowerment] explode all trees | 48 |
| #17 | #11 or #12 or #13 or #14 or #15 or #16 | 40997 |
| #18 | #10 and #17 | 2420 |
| #19 | MeSH descriptor: [Anxiety] explode all trees | 9165 |
| #20 | MeSH descriptor: [Anxiety Disorders] explode all trees | 7824 |
| #21 | MeSH descriptor: [Depression] explode all trees | 14069 |
| #22 | MeSH descriptor: [Quality of Life] explode all trees | 28904 |
| #23 | (anxiety or depression):ti,ab | 101554 |
| #24 | quality of life:ti,ab | 125719 |
| #25 | #19 or #20 or #21 or #22 or #23 or #24 | 217524 |
| #26 | #18 and #25 | 684 |

| **PSCYINFO** | | |
| --- | --- | --- |
| **#** | **Consulta** | **Resultados** |
| S27 | S19 AND S26 | 138 |
| S26 | S20 OR S21 OR S22 OR S23 OR S24 OR S25 | 509,5 |
| S25 | TI "Quality of Life" OR AB "Quality of Life" | 77,87 |
| S24 | TI "Quality of Life" OR AB "Quality of Life" | 77,87 |
| S23 | DE "Quality of Life" | 62,821 |
| S22 | TI ( (Depression or depressive disorder or anxiety or anxiety disorder) ) OR AB ( (Depression or depressive disorder or anxiety or anxiety disorder) ) | 417,047 |
| S21 | DE "Depression (Emotion)" | 26,61 |
| S20 | DE "Anxiety" OR DE "Anxiety Disorders" | 116,131 |
| S19 | S10 AND S18 | 570 |
| S18 | S11 OR S12 OR S13 OR S14 OR S15 OR S16 OR S17 | 88,235 |
| S17 | TI empowerment OR AB empowerment | 17,422 |
| S16 | ( (self efficacy or self-efficacy or patient participation or client participation) ) OR AB ( (self efficacy or self-efficacy or patient participation or client participation) ) | 68,687 |
| S15 | DE "Empowerment" | 9,038 |
| S14 | DE "Client Participation" | 2,851 |
| S13 | DE "Self-Efficacy" | 26,6 |
| S12 | self efficacy | 58,345 |
| S11 | TI ( ((patient* or adult* or client* or participant* or individual*) N3 empower*) ) OR AB ( ((patient* or adult* or client* or participant* or individual*) N3 empower*) ) | 4,186 |
| S10 | S6 NOT S9 | 6,636 |
| S9 | S7 OR S8 | Mostrar |
| S8 | TX Diabetes insipidus | 389 |
| S7 | DE "Diabetes Insipidus" | 230 |
| S6 | S1 OR S2 OR S3 OR S4 OR S5 |  |
| S5 | TI ( (((late or adult* or matur* or slow or stabl*) N3 onset) and diabet* ) OR AB ( (((late or adult* or matur* or slow or stabl*) N3 onset) and diabet* ) |  |
| S4 | TI ( (MODY or NIDDM or T2D*) ) OR AB ( (MODY or NIDDM or T2D*) ) |  |
| S3 | TI ( ((typ? 2 or typ? II or typ?2 or typ?II) N3 diabet*) ) OR AB ( ((typ? 2 or typ? II or typ?2 or typ?II) N3 diabet*) ) |  |
| S2 | TI ( (non insulin* depend* or noninsulin* depend* or noninsulin?depend* or non insulin?depend*) ) OR AB ( (non insulin* depend* or noninsulin* depend* or noninsulin?depend* or non insulin?depend*) ) |  |
| S1 | DE "Type 2 Diabetes" OR DE "Blood Sugar" |  |
