# Supplementary material for: The relationship between patient empowerment and related constructs, affective symptoms and quality of life in patients with type 2 diabetes: a systematic review and meta-analysis
Source: Front Public Health. 2023 Apr 17;11:1118324. doi: 10.3389/fpubh.2023.1118324 (PMC10150112; doi:10.3389/fpubh.2023.1118324)

Supplementary Material 3

The Relationship Between Patient Empowerment and Related Constructs, Affective Symptoms and Quality of Life in Patients with Type 2 Diabetes: A Systematic Review and Meta-Analysis

Andrea Duarte-Díaz^1,2,3,4^, Lilisbeth Perestelo-Pérez^3,4,5^*, Amado Rivero-Santana^1,3,4^, Wenceslao Peñate^2^, Yolanda Álvarez-Pérez^1,3,4^, Vanesa Ramos-García^1,3,4^, Himar González-Pacheco^1,3,4^, Libertad Goya-Arteaga^6^, Miriam de Bonis-Braun^6^, Silvia González-Martín^6^, Yolanda Ramallo-Fariña^1,3,4^, Carme Carrion^3,7^ and Pedro Serrano-Aguilar^3,4,5^

*** Correspondence:** Lilisbeth Perestelo-Pérez: lilisbeth.presteloperez@sescs.es

# Supplementary Material 3. Quality assessment of the included studies

1. Cross-sectional studies

| **Author, year** | **Joana Briggs Institute checklist for analytical cross-sectional studies** | | | | | | | |
| --- | --- | --- | --- | --- | --- | --- | --- | --- |
|  | **1. Were the criteria for inclusion in the sample clearly defined?** | **2. Were the study subjects and the setting described in detail?** | **3. Was the exposure measured in a valid and reliable way?** | **4. Were objective, standard criteria used for measurement of the condition?** | **5. Were confounding factors identified?** | **6. Were strategies to deal with confounding factors stated?** | **7. Were the outcomes measured in a valid and reliable way?** | **8. Was appropriate statistical analysis used?** |
| Ababio, 2017 | Yes | Yes | Yes | Yes | Yes | Unclear | Yes | Yes |
| Abdelgaffar, 2020 | Unclear | Yes | Yes | Yes | No | No | Yes | Yes |
| Al Amer, 2016 | Yes | Yes | Yes | Yes | No | No | Yes | Yes |
| Al Dwaikat, 2020 | Yes | Yes | Yes | Yes | No | No | Yes | Yes |
| Alipour, 2012 | Yes | Yes | Yes | Yes | No | No | Yes | Yes |
| Alzubaidi, 2022 | Yes | Yes | Yes | Yes | No | No | Yes | Yes |
| Anderson, 2016 | Yes | Yes | Yes | Yes | Yes | Yes | Yes | Yes |
| Aoto, 2019 | Yes | Yes | Yes | Yes | No | No | Yes | Yes |
| Arvanitis, 2020 | Yes | Yes | Yes | Yes | Yes | Yes | Yes | Yes |
| Azadbakht, 2021 | Yes | Yes | Yes | Yes | Yes | Yes | Yes | Yes |
| Azami, 2020 | Yes | Yes | Yes | Yes | No | No | Yes | Yes |
| Chao, 2005 | Yes | Yes | Yes | Yes | Yes | Yes | Yes | Yes |
| Cherrington, 2020 | Yes | Yes | Yes | Yes | Yes | Yes | Yes | Yes |
| Chew, 2018 | Yes | Yes | Yes | Yes | Yes | Yes | Yes | Yes |
| Ching, 2020 | No | Yes | Yes | Yes | Yes | Unclear | Yes | Yes |
| Coffman, 2008 | No | Yes | Yes | Yes | Yes | Yes | Yes | Yes |
| Devarajooh, 2017 | Yes | Yes | Yes | Yes | Yes | Unclear | Yes | Yes |
| Emery, 2021 | Yes | Yes | Yes | Yes | Yes | Yes | Yes | Yes |
| Fereydouni, 2022 | Yes | Yes | Yes | Yes | Yes | Yes | Yes | Yes |
| Gonzalez, 2015 | Yes | Yes | Yes | Yes | Yes | Yes | Yes | Yes |
| Hernández, 2016 | Yes | Yes | Yes | Yes | Yes | Yes | Yes | Yes |
| Hernández-Tejada, 2012 | Unclear | Yes | Yes | Unclear | Yes | Yes | Yes | Yes |
| Huang, 2020 | Yes | Yes | Yes | Yes | Yes | Yes | Yes | Yes |
| Huayanai, 2021 | Yes | Yes | Yes | Yes | Yes | Yes | Yes | Yes |
| Indelicato, 2017 | Yes | Yes | Yes | Yes | Yes | Yes | Yes | Yes |
| Jahanlou, 2011 | No | Yes | Yes | Yes | Yes | No | Yes | Yes |
| Ji, 2020 | Yes | Yes | Yes | Yes | Yes | Yes | Yes | Yes |
| Ji, 2021 | Yes | Yes | Yes | Yes | Yes | Yes | Yes | Yes |
| Kato, 2016 | Yes | Yes | Yes | Yes | Yes | Yes | Yes | Yes |
| Kav, 2017 | Yes | Yes | Yes | Yes | Yes | Unclear | Yes | Yes |
| Kim, 2019 | Yes | Yes | Yes | Yes | Yes | Yes | Yes | Yes |
| Kobling, 2020 | Yes | Yes | Yes | Yes | No | No | Yes | Yes |
| Lin, 2017 | Yes | Yes | Yes | Yes | Yes | Yes | Yes | Yes |
| Lin, 2020 | Yes | Yes | Yes | Yes | Yes | No | Yes | Yes |
| Matteucci, 2003 | No | Yes | Yes | Yes | Yes | Yes | Yes | Yes |
| Messina, 2018 | Yes | Yes | Yes | Yes | Yes | No | Yes | Yes |
| Oliveira, 2016 | Yes | Yes | Yes | Yes | Yes | Yes | Yes | Yes |
| Oviedo-Gómez, 2007 | Yes | Yes | Yes | Yes | Yes | No | Yes | Yes |
| Padgett, 1991 | Yes | Yes | Yes | Yes | Yes | Yes | Yes | Yes |
| Park, 2012 | Yes | Yes | Yes | Yes | Yes | Yes | Yes | Yes |
| Paschalides, 2014 | Yes | Yes | Yes | Yes | Yes | Yes | Yes | Yes |
| Pisanti, 2005 | No | Yes | Yes | Yes | Yes | Yes | Yes | Yes |
| Rusni, 2020 | No | Yes | Unclear | Yes | No | No | Unclear | Unclear |
| Sacco, 2007 | Yes | Yes | Yes | Yes | Yes | Unclear | Yes | Yes |
| Sacco, 2005 | Yes | Yes | Yes | Yes | Yes | No | Yes | Yes |
| Samuel-Hodge, 2013 | Yes | Yes | Yes | Yes | Yes | Yes | Yes | Yes |
| Sari, 2021 | Yes | Yes | Yes | Yes | Yes | Yes | Yes | Yes |
| Sit, 2022 | Yes | Yes | Yes | Yes | Yes | Yes | Yes | Yes |
| Simonsen, 2021 | Yes | Yes | Yes | Yes | Yes | Yes | Yes | Yes |
| Song, 2014 | Unclear | Yes | Yes | Yes | Yes | Yes | Yes | Yes |
| Suhaimi, 2022 | Yes | Yes | Yes | Yes | No | No | Yes | Yes |
| Sympa, 2018 | Yes | Yes | Yes | Yes | Yes | No | Yes | Yes |
| Tol, 2015 | Yes | Yes | Yes | Yes | Yes | Yes | Yes | Yes |
| Walker, Gebregziabher, 2014 | Yes | Yes | Yes | Yes | Yes | Yes | Yes | Yes |
| Walker, Smalls, 2014 | Yes | Yes | Yes | Yes | Yes | Yes | Yes | Yes |
| Wang, 2011 | Yes | Yes | Yes | Yes | Yes | Yes | Yes | Yes |
| Williams, 2005 | Yes | Yes | Yes | Yes | No | No | Yes | Yes |
| Winahyu, 2019 | Yes | Yes | Yes | Yes | No | No | Yes | Yes |
| Wu, 2013 | Yes | Yes | Yes | Yes | No | No | Yes | Yes |
| Yang, 2016 | Yes | Yes | Yes | Yes | Yes | Yes | Yes | Yes |
| Zhu, 2016 | Yes | Yes | Yes | Yes | Yes | Yes | Yes | Yes |

1. Prospective studies

| **NIH Quality Assessment Tool for Observational Cohort Studies** | **Author, year** | | | | |
| --- | --- | --- | --- | --- | --- |
|  | **Duarte-Díaz, 2022** | **Hsu, 2021** | **Latham, 2009** | **Latham, 2013** | **Rao, 2020** |
| **1. Was the research question or objective in this paper clearly stated?** | Yes | Yes | Yes | Yes | Yes |
| **2. Was the study population clearly specified and defined?** | Yes | Yes | Yes | Yes | Yes |
| **3. Was the participation rate of eligible persons at least 50%?** | Yes | Yes | Yes | Yes | No |
| **4. Were all the subjects selected or recruited from the same or similar populations (including the same time period)? Were inclusion and exclusion criteria for being in the study prespecified and applied uniformly to all participants?** | Yes | Yes | No | No | Yes |
| **5. Was a sample size justification, power description, or variance and effect estimates provided?** | No | Yes | Yes | No | Yes |
| **6. For the analyses in this paper, were the exposure(s) of interest measured prior to the outcome(s) being measured?** | Yes | Yes | Yes | Yes | Yes |
| **7. Was the timeframe sufficient so that one could reasonably expect to see an association between exposure and outcome if it existed?** | Yes | Yes | Yes | Yes | No |
| **8. For exposures that can vary in amount or level, did the study examine different levels of the exposure as related to the outcome (e.g., categories of exposure, or exposure measured as continuous variable)?** | Yes | Yes | Yes | Yes | Yes |
| **9. Were the exposure measures (independent variables) clearly defined, valid, reliable, and implemented consistently across all study participants?** | Yes | Yes | Yes | Yes | Yes |
| **10. Was the exposure(s) assessed more than once over time?** | Yes | No | No | No | No |
| **11. Were the outcome measures (dependent variables) clearly defined, valid, reliable, and implemented consistently across all study participants?** | Yes | Yes | Yes | Yes | Yes |
| **12. Were the outcome assessors blinded to the exposure status of participants?** | NA | NA | NA | NA | NA |
| **13. Was loss to follow-up after baseline 20% or less?** | Yes | No | Yes | Yes | No |
| **14. Were key potential confounding variables measured and adjusted statistically for their impact on the relationship between exposure(s) and outcome(s)?** | Yes | Yes | Yes | Yes | NR |

1. Pre-post study

| **NIH Quality Assessment Tool for pre-post Studies** | **Author, year** |
| --- | --- |
|  | **Clarke, 2011** |
| **1. Was the study question or objective clearly stated?** | Yes |
| **2. Were eligibility/selection criteria for the study population prespecified and clearly described?** | No |
| **3. Were the participants in the study representative of those who would be eligible for the test/service/intervention in the general or clinical population of interest?** | CD |
| **4. Were all eligible participants that met the prespecified entry criteria enrolled?** | CD |
| **5. Was the sample size sufficiently large to provide confidence in the findings?** | CD |
| **6. Was the test/service/intervention clearly described and delivered consistently across the study population?** | Yes |
| **7. Were the outcome measures prespecified, clearly defined, valid, reliable, and assessed consistently across all study participants?** | Yes |
| **8. Were the people assessing the outcomes blinded to the participants' exposures/interventions?** | CD |
| **9. Was the loss to follow-up after baseline 20% or less? Were those lost to follow-up accounted for in the analysis?** | Yes |
| **10. Did the statistical methods examine changes in outcome measures from before to after the intervention? Were statistical tests done that provided p values for the pre-to-post changes?** | Yes |
| **11. Were outcome measures of interest taken multiple times before the intervention and multiple times after the intervention (i.e., did they use an interrupted time-series design)?** | No |
| **12. If the intervention was conducted at a group level (e.g., a whole hospital, a community, etc.) did the statistical analysis take into account the use of individual-level data to determine effects at the group level?** | Yes |
| **CD:** cannot determine; **N/A:** not applicable; **NR:** not reported | |

1. Randomized controlled trials

**
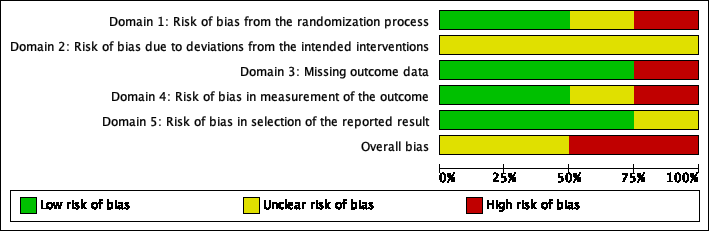
**


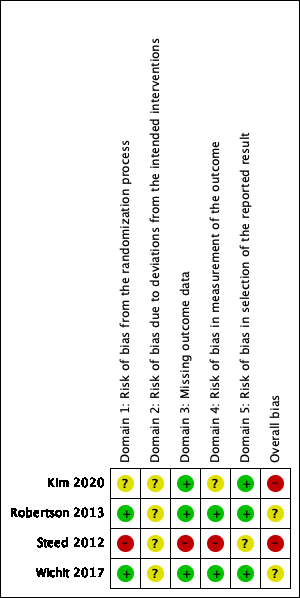

Supplement: Supplementary file 3 [file Data_Sheet_3.docx]
