# Supplementary material for: The relationship between patient empowerment and related constructs, affective symptoms and quality of life in patients with type 2 diabetes: a systematic review and meta-analysis
Source: Front Public Health. 2023 Apr 17;11:1118324. doi: 10.3389/fpubh.2023.1118324 (PMC10150112; doi:10.3389/fpubh.2023.1118324)
Supplement: Supplementary file 4 [file Data_Sheet_4.docx]

Supplementary Material 4

The Relationship Between Patient Empowerment and Related Constructs, Affective Symptoms and Quality of Life in Patients with Type 2 Diabetes: A Systematic Review and Meta-Analysis

Andrea Duarte-Díaz^1,2,3,4^, Lilisbeth Perestelo-Pérez^3,4,5^*, Amado Rivero-Santana^1,3,4^, Wenceslao Peñate^2^, Yolanda Álvarez-Pérez^1,3,4^, Vanesa Ramos-García^1,3,4^, Himar González-Pacheco^1,3,4^, Libertad Goya-Arteaga^6^, Miriam de Bonis-Braun^6^, Silvia González-Martín^6^, Yolanda Ramallo-Fariña^1,3,4^, Carme Carrion^3,7^ and Pedro Serrano-Aguilar^3,4,5^

*** Correspondence:** Lilisbeth Perestelo-Pérez: lilisbeth.presteloperez@sescs.es

# Supplementary Material 4. Sensitivity analyses

**Supplementary File 4-A.** Leave-one-out approach showing the influence of each individual study on the pooled correlation coefficient between empowerment and anxiety

**Supplementary File 4-B.** Leave-one-out approach showing the influence of each individual study on the pooled correlation coefficient between empowerment and depression

**Supplementary File 4-C.** Leave-one-out approach showing the influence of each individual study on the pooled correlation coefficient between empowerment and distress

**Supplementary File 4-D.** Leave-one-out approach showing the influence of each individual study on the pooled correlation coefficient between empowerment and general QoL

**Supplementary File 4-E.** Leave-one-out approach showing the influence of each individual study on the pooled correlation coefficient between empowerment and mental QoL

**Supplementary File 4-F.** Leave-one-out approach showing the influence of each individual study on the pooled correlation coefficient between empowerment and physical QoL
