# Supplementary material for: The relationship between patient empowerment and related constructs, affective symptoms and quality of life in patients with type 2 diabetes: a systematic review and meta-analysis
Source: Front Public Health. 2023 Apr 17;11:1118324. doi: 10.3389/fpubh.2023.1118324 (PMC10150112; doi:10.3389/fpubh.2023.1118324)
Supplement: Supplementary file 5 [file Data_Sheet_5.docx]

Supplementary Material 5

The Relationship Between Patient Empowerment and Related Constructs, Affective Symptoms and Quality of Life in Patients with Type 2 Diabetes: A Systematic Review and Meta-Analysis

Andrea Duarte-Díaz^1,2,3,4^, Lilisbeth Perestelo-Pérez^3,4,5^*, Amado Rivero-Santana^1,3,4^, Wenceslao Peñate^2^, Yolanda Álvarez-Pérez^1,3,4^, Vanesa Ramos-García^1,3,4^, Himar González-Pacheco^1,3,4^, Libertad Goya-Arteaga^6^, Miriam de Bonis-Braun^6^, Silvia González-Martín^6^, Yolanda Ramallo-Fariña^1,3,4^, Carme Carrion^3,7^ and Pedro Serrano-Aguilar^3,4,5^

*** Correspondence:** Lilisbeth Perestelo-Pérez: lilisbeth.presteloperez@sescs.es

**Supplementary Material 5.** **Subgroup analyses**

**Supplementary File 5-A.** Relationship between patient empowerment and depression

| **Subgroup** | **k** | **r** | **95%CI** | **I^2^** | **Test for subgroup differences** |
| --- | --- | --- | --- | --- | --- |
| **Exposure**  Activation  Empowerment  Perceived control  Self-efficacy | 2  5  2  23 | -0.34  -0.17  -0.32  -0.32 | -0.43, -0.26  -0.17, -0.36  -0.32, -0.39  -0.32, -0.36 | 0.00%  92.73%  0.00%  55.49% | Q = 2.61, p = 0.455 |
| **Exposure**  Empowerment  Indicators | 5  27 | -0.17  -0.32 | -0.36, 0.03  -0.35, -0.28 | 92.73%  47.67% | Q = 2.14, p = 0.143 |

**Supplementary File 5-B.** Relationship between patient empowerment and distress

| **Subgroup** | **k** | **r** | **95%CI** | **I^2^** | **Test for subgroup differences** |
| --- | --- | --- | --- | --- | --- |
| **Exposure**  Empowerment  Self-efficacy | 3  16 | -0.26  -0.34 | -0.44, -0.09  -0.42, -0.26 | 92.14%  80.84% | Q = 0.60, p = 0.439 |
| **Outcome**  General distress  Specific distress | 3  16 | -0.19  -0.34 | -0.30, -0.08  -0.42, -0.27 | 25.10%  87.13% | Q = 4.69, p = 0.030 |

**Supplementary File 5-C.** Relationship between patient empowerment and general quality of life

| **Subgroup** | **k** | **r** | **95%CI** | **I^2^** | **Test for subgroup differences** |
| --- | --- | --- | --- | --- | --- |
| **Exposure**  Empowerment  Self-efficacy | 5  17 | 0.27  0.35 | 0.13, 0.42  0.27, 0.43 | 87.35%  80.33% | Q = 0.72, p = 0.396 |
| **Type of association**  Cross-sectional  Longitudinal | 18  4 | 0.34  0.26 | 0.26, 0.43  0.19, 0.32 | 85.04%  0.01% | Q = 2.54, p = 0.111 |

**Supplementary File 5-D.** Relationship between patient empowerment and mental quality of life

| **Subgroup** | **k** | **r** | **95%CI** | **I^2^** | **Test for subgroup differences** |
| --- | --- | --- | --- | --- | --- |
| **Exposure**  Perceived control  Self-efficacy | 2  7 | 0.47  0.17 | 0.22, 0.71  0.04, 0.31 | 82.13%  87.16% | Q = 4.35, p = 0.037 |

**Supplementary File 5-E.** Relationship between patient empowerment and physical quality of life

| **Subgroup** | **k** | **r** | **95%CI** | **I^2^** | **Test for subgroup differences** |
| --- | --- | --- | --- | --- | --- |
| **Exposure**  Perceived control  Self-efficacy | 2  7 | 0.26  0.08 | 0.08, 0.50  -0.00, 0.17 | 67.16%  56.32% | Q = 3.47, p = 0.063 |
